# Insights into targeted ferroptosis in mechanisms, biology, and role of Alzheimer's disease: an update

**DOI:** 10.3389/fnagi.2025.1587986

**Published:** 2025-07-21

**Authors:** Bingyuan Zhou, Jing Li, Anqi Wu, Xuewei Wang, Le Cheng, Gaoshang Yang, Dahong Gao, Caifeng Zhu

**Affiliations:** ^1^The First Clinical Medical School, Anhui University of Traditional Chinese Medicine, Hefei, China; ^2^Fourth Department of Encephalopathy, Second Affiliated Hospital of Anhui University of Traditional Chinese Medicine, Hefei, Anhui, China; ^3^Medical Department, Second Affiliated Hospital of Anhui University of Traditional Chinese Medicine, Hefei, Anhui, China; ^4^Third Department of Geriatrics, Second Affiliated Hospital of Anhui University of Traditional Chinese Medicine, Hefei, Anhui, China; ^5^Clinical Research Institute of Acupuncture and Moxibustion, Anhui Academy of Traditional Chinese Medicine Sciences, Hefei, Anhui, China

**Keywords:** Alzheimer's disease, ferroptosis, mechanisms, update, biology

## Abstract

Ferroptosis is a newly discovered form of programmed cell death, primarily caused by an imbalance between iron-dependent oxidative damage and antioxidant defense mechanisms within the cell. It differs from previously reported forms of cell death, such as apoptosis, necrosis, and autophagy, in terms of morphology, biochemistry, and genetics. Alzheimer's disease (AD) is the most common neurodegenerative disorder, characterized by pathological features including neurofibrillary tangles (NFTs), senile plaques (SPs), and abnormal iron deposition, suggesting that ferroptosis may be involved in its disease progression. Although recent studies have made significant progress, the mechanisms underlying neuronal ferroptosis in AD remain incompletely understood. This review, based on elucidating the process and regulatory mechanisms of cellular ferroptosis, explores, and supplements the correlation between iron overload and redox imbalance with the main pathological mechanisms of AD, providing new insights for the treatment of AD and the development of new drugs.

## 1 Introduction

Alzheimer's disease (AD) is a neurodegenerative disorder triggered by multiple etiologies, characterized by insidious onset and slow progression. It is the most common type of dementia. Currently, there are approximately 50 million cases of AD worldwide, and by 2050, the prevalence of AD is projected to triple globally. This substantial patient population imposes a heavy burden on society and the economy (Rostagno, 2022; Scheltens et al., 2021). AD is primarily characterized by the gradual loss of cognitive functions, accompanied by the deterioration of language, reasoning, spatial awareness, and even mobility, ultimately leading to the loss of daily living abilities (Beata et al., 2023). The main pathological features of AD include the accumulation of β-amyloid (Aβ) forming extracellular senile plaques (SPs) and the hyperphosphorylation of the microtubule-associated protein tau (MAPT) forming intracellular neurofibrillary tangles (NFTs) (Graff-Radford et al., 2021). Since the pathogenesis of AD remains unclear, there are currently no effective means to prevent the onset of AD or slow its progression. Recent studies on the molecular mechanisms of ferroptosis in neurodegenerative diseases have shown that it may become a potential therapeutic target for such diseases. Ferroptosis is an iron-dependent, lipid peroxidation-driven form of cell death that is associated with the pathogenesis of AD and is involved in the disease process (Wu et al., 2023). Several studies (Ansari and Scheff, 2010; Cheng et al., 2021; Lill and Freibert, 2020) have observed biochemical and morphological characteristics of ferroptosis in the brains of AD patients or mice, including iron metabolism imbalance, glutathione (GSH) degradation, inactivation of glutathione peroxidase 4 (GPX4) leading to increased reactive oxygen species (ROS), lipid peroxidation, and mitochondrial abnormalities. In the pathological environment of AD, increased neuronal iron uptake and impaired iron efflux lead to iron metabolism imbalance, manifested by elevated iron levels in the hippocampus, cortex, and basal ganglia regions of the brains of AD patients. Iron deposition in AD lesions promotes the accumulation of Aβ plaques to form SPs (Pinheiro and Faustino, 2019) and the hyperphosphorylation of tau protein to form NFTs (Spotorno et al., 2020). Conversely, the aggregation of Aβ plaques and the hyperphosphorylation of tau protein inhibit iron efflux, leading to further iron accumulation and creating a vicious cycle in AD pathogenesis. Studies (Gong et al., 2024; Li X. et al., 2023) have shown that regulating ferroptosis can improve AD pathology. This review focuses on the unique characteristics of ferroptosis, systematically summarizes its role and mechanisms in the course of AD, and aims to provide new strategies for the basic treatment and clinical research of AD.

## 2 Overview of ferroptosis

**Figure 1:**
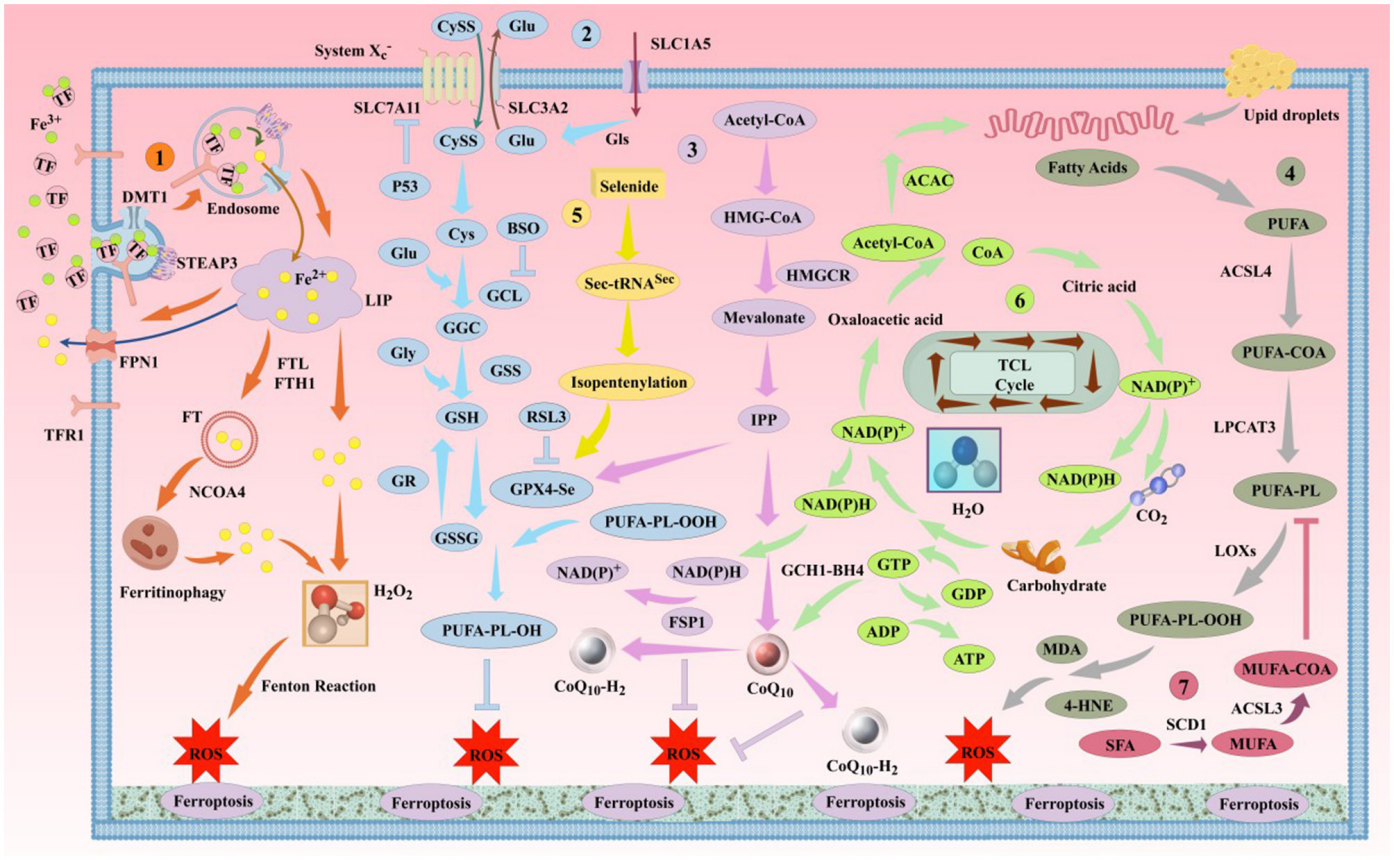
The classic mechanism of ferroptosis. Ferroptosis is primarily driven by iron-dependent lipid peroxidation. This process involves several pathways, including the iron metabolism pathway, the Cys-GSH-GPX4 pathway, the mevalonate pathway, and the lipid metabolism pathway, among others. DMT1, divalent metal ion transporter 1; STEAP3, prostate 6 transmembrane epithelial antigen 3; NCOA4, nuclear receptor coactivator 4; SLC7A11, solute carrier family 7 member 11; SLC3A2, solute carrier family 3 member 2; SLC1A5, solute carrier family 1 member 5; GLS, glutaminase; P53, tumor protein 53; BSO, buthionine sulfoximine; GCL, glutamate-cysteine ligase; GGC, γ-L-glutamyl-L-cysteine; GSS, glutathione synthetase; GR, glutathione reductase; GSSG, reduced glutathione; HMGCR, 3-hydroxy-3-methylglutaryl-CoA reductase; IPP, isoprenoid diphosphate; ACAC, acetyl-CoA carboxylase; LPCAT3, lysophosphatidylcholine acyltransferase 3; LOX, lipoxygenase; ACSL3, acyl-CoA synthetase long-chain family member 3; SCD1, stearoyl-CoA desaturase 1; BH4, tetrahydrobiopterin; GCH1, human GTP cyclohydrolase 1.

**Figure 2:**
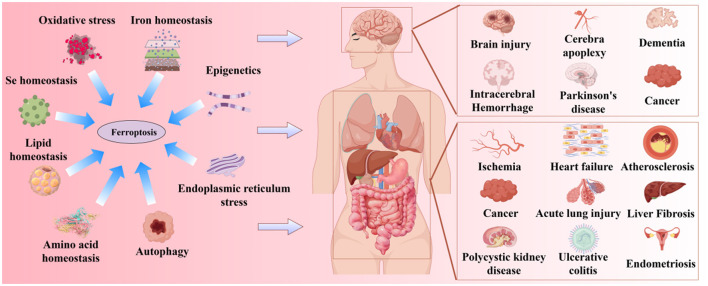
Ferroptosis is related to the occurrence of multiple major diseases.

Ferroptosis was first proposed by Stockwell in 2012 (Dixon et al., 2012). It is a novel form of iron-dependent cell death, characterized by the excessive accumulation of ROS produced by iron metabolism and lipid peroxidation products produced by lipid metabolism, as well as the abnormality of the GSH metabolic pathway caused by amino acid metabolism disorders, which is different from apoptosis, necrosis, and autophagy in morphology and biochemistry (Bruedigam et al., 2024; Xie et al., 2016). Morphologically, the typical features of ferroptosis include cell brightness, reduced volume, intact cell membrane with increased density but compromised integrity, normal-sized and intact cell nuclei, and recent studies have found that the cell nuclei can appear electronlucent (Miyake et al., 2020; Ou et al., 2022; Riegman et al., 2020). In addition, mitochondria undergo condensation, reduced or disappeared cristae, increased mitochondrial potential, outer membrane rupture, no chromatin condensation, no apoptotic bodies, and no autophagic vesicles (Lei et al., 2019; Qin et al., 2021). Biochemically, the depletion of intracellular GSH and the inactivation of GPX4 lead to cell ferroptosis (Maiorino et al., 2018). Ferroptosis can be induced by two pathways: exogenous and endogenous. The exogenous pathway consists of the activation of cell membrane transporters for transferrin (TF) and lactoferrin (LF) or the inhibition of cystine/glutamate (CySS/Glu) antiporters, while the endogenous pathway is the inhibition of GPX4 activation within cells (Li J. et al., 2020; Tang et al., 2021). Its core molecular mechanism is the imbalance between oxidative damage and antioxidant defense (Figure 1). Increasing evidence suggests that ferroptosis is closely related to the development of several important diseases, including neurodegenerative diseases, cardiomyopathies, metabolic liver diseases, and respiratory disorders (Figure 2). Further exploration of the specific mechanisms linking ferroptosis to disease progression will deepen our understanding of these significant diseases and provide new strategies for more precise prevention and treatment options for patients.

## 3 Biological regulatory mechanisms of ferroptosis

### 3.1 Classic biological regulatory mechanisms of ferroptosis

#### 3.1.1 Iron homeostasis

Iron is an essential trace element for the human body, and its deficiency or excess can cause damage to cells and even the entire body. Dietary ferric ions (Fe3^+^) are reduced to ferrous ions (Fe^2^^+^) in the small intestine and then enter the bloodstream through the intestinal basolateral membrane iron transporter (FPN1, SLC11A3) (Outten and Theil, 2009). During transport, the iron transport auxiliary protein (HEPH) oxidizes Fe^2^^+^ to Fe3^+^ and binds it to TF, forming the TF-Fe3^+^ complex, which enters cells via the specific receptor TFR1 (Cheng et al., 2004; Gao et al., 2015). In the acidic environment of endosomes, Fe3^+^ is released from TF and reduced to Fe^2^^+^ by STEAP3, and then DMT1 (SLC11A2) or zinc iron regulatory protein family 8/14 (ZIP8/14) releases Fe^2^^+^ into the cytoplasmic labile iron pool (LIP) (Bogdan et al., 2016; Ohgami et al., 2005). In the LIP, Fe^2^^+^ combines with ferritin light chain polypeptide (FTL) and ferritin heavy chain polypeptide 1 (FTH1) to form an iron storage protein complex (Torti and Torti, 2013). FPN1 oxidizes excess Fe^2^^+^ to Fe3^+^ and transports it out of the cell, strictly controlling intracellular iron homeostasis.

Imbalances in iron metabolism, especially iron overload, are important causes of ferroptosis. The two main sources of intracellular Fe^2^^+^ accumulation during ferroptosis are: (1) TF binds to TFR and enters the cell, where the low pH value promotes the release of Fe3^+^ from TF, which is then reduced to Fe^2^^+^ in the cytoplasm; (2) LIP ferritin (FT) can be autophagocytosed and degraded by NCOA4-mediated autophagy, releasing large amounts of Fe^2^^+^. Pathologically, the accumulation of free Fe^2^^+^ in cells promotes the production of hydroxyl radicals that cause oxidative damage to DNA, proteins, and membrane lipids through Fenton reactions or iron-catalyzed Haber-Weiss reactions; on the other hand, Fe^2^^+^ can act as a cofactor for various metabolic enzymes, enhancing their activity and promoting the generation of ROS. Therefore, intracellular iron deposition generates ROS and causes oxidative stress (OS), promoting lipid peroxidation and ferroptosis (Cao and Dixon, 2016; Duan et al., 2017; Yu et al., 2019).

#### 3.1.2 Oxidative stress

Sies (1991) first defined OS as “the accumulation of ROS and cellular damage caused by the imbalance between pro-oxidant and antioxidant systems.” ROS are a group of chemically reactive molecules and ions with high oxidizing activity, including superoxide anions, peroxides, and free radicals, which play important roles in cell signaling and tissue homeostasis (Ferreira et al., 2018). In neurodegenerative diseases, mitochondria are the main source of ROS in cells undergoing ferroptosis (Wu C. et al., 2018). In neurodegenerative diseases, mitochondria are the main source of ROS in cells undergoing ferroptosis (Guerrero-Hue et al., 2019; Sakellariou et al., 2014). The body has a ROS clearance system to maintain internal homeostasis, including enzymatic and non-enzymatic antioxidants, which can eliminate excess ROS and keep ROS levels within a certain range, playing important roles in anti-infection, tumor suppression, and intracellular signaling (Michaeloudes et al., 2022). Tumor protein 53 (p53) can directly regulate cellular metabolism shifts by promoting mitochondrial oxidative phosphorylation (OXPHOS), leading to increased ROS production and is a positive regulator of ferroptosis. However, p53 can protect cells by eliminating ROS under mild stress. The cell reactions mediated by p53-induced ROS production still need further research (Zhang et al., 2018).

In addition, compounds targeting mitochondrial nitric oxide radicals can effectively inhibit ferroptosis in various cells, indicating that mitochondrial lipid peroxidation plays a key role in ferroptotic OS (Krainz et al., 2016). When the body is in a pathological state, changes in cell membrane phospholipid reconstruction and increased free iron concentration promote the occurrence of lipid peroxidation chain reactions, leading to increased production of specific lipid peroxides (LPO). The insufficient function of the antioxidant system leads to reduced clearance of LPO, and the dynamic imbalance of ROS generation and clearance leads to the accumulation of free radicals, thereby damaging the structure and function of DNA, proteins, and lipids, triggering OS, which is a direct inducer of ferroptosis (Latunde-Dada, 2017; Poljsak et al., 2013; Stockwell et al., 2017). The lipid peroxidation process that induces ferroptosis can occur on the outer membranes of the cell membrane, mitochondria, endoplasmic reticulum, and lipid bodies. Mitochondria, as one of the main sources of endogenous ROS, are sensitive to lipid peroxidation. Polyunsaturated fatty acid (PUFA)-containing phosphatidylethanolamine (PE) is more susceptible to lipid peroxidation. The accumulation of PE-AA-OOH and PE-AdA-OOH on the outer membrane of mitochondria can lead to increased membrane permeability, mitochondrial swelling, and eventual outer membrane rupture. Riegman et al. (2020) found that cell ferroptosis involves osmotic effects and is accompanied by cell swelling, membrane pore formation, and wave-like propagation to surrounding cells, occurring before cell rupture. This suggests that mitochondrial fragmentation during ferroptosis may be due to changes in osmotic pressure between the inner and outer mitochondrial membranes caused by lipid peroxidation. Therefore, OS runs through the entire pathological process of ferroptosis, and ferroptosis can also serve as a new target to regulate OS and provide protection.

#### 3.1.3 Selenium homeostasis

Selenium (Se) is an essential trace element for humans and plays important biological effects in life activities, including antioxidant, immune regulation, detoxification of toxins, and regulation of signaling pathways. After Se is absorbed into the body, it is regulated by key hepatic enzymes and other regulatory factors, and then synthesized into selenoproteins (Gammelgaard et al., 2012). By regulating Se content, the expression or function of selenoproteins can be affected, thereby indirectly regulating the occurrence of ferroptosis. Selenoproteins, as the main mediators of Se physiological and toxic effects, exert different biological functions and participate in multiple molecular pathways of ferroptosis. Among them, GPX4 is a unique member of selenoproteins and is the only enzyme in the body that can reduce peroxides to alcohols, participating in the body's oxidative defense system (Schnurr et al., 1996). GPX4 can bind to lipids or proteins and reduce lipid peroxides in cell membranes, inhibiting lipid peroxidation and LPO-induced cell death (Brigelius-Flohe and Maiorino, 2013). The inhibition of the CySS/Glu antiporter system reduces GSH synthesis and GPX4 inactivation, leading to the accumulation of polyunsaturated fatty acids (PUFA) and ROS, and causing membrane integrity damage and ferroptosis (Friedmann Angeli and Conrad, 2018; Mortensen et al., 2023). The mRNA sequence of GPX4 contains a selenocysteine insertion sequence (SECIS) element in the3′ untranslated region, which encodes UGA-Sec in the active site through the UGA codon (Berry et al., 1991; Yang and Stockwell, 2016). The mevalonate pathway (MVA) directly produces isoprenoid diphosphate (IPP), which promotes the maturation of Ser-Sec tRNA and is a necessary condition for the binding of Sec and GPX4 (Yang and Stockwell, 2016). Se can increase the density of ribosomes downstream of the UGA-Sec codon or partially increase the methylation degree of Sec-tRNA[Ser]SecUm34 through various pathways, thereby improving the binding efficiency of Sec (Howard et al., 2013). The small molecule compound RSL3 can also bind to the Sec in the active site of GPX4, directly inhibiting the antioxidant activity of GPX4 and inducing ferroptosis (Yang et al., 2014; Yang and Stockwell, 2008).

In addition to GPX4, Se is also found as a key component of other selenoproteins and maintains their antioxidant functions. Thioredoxin reductase (TrxR) family (containing Cys or Sec residues in the active site motif) (Holubiec et al., 2022), such as TXNRD1, is also an important selenoprotein containing Se. Wang H. et al. (2023) found that selenium supplementation can increase the activity and expression levels of TXNRD1, reduce ROS levels, and enhance cell viability and proliferation. Methionine (Met) is one of the most easily oxidized sulfur-containing amino acids, and the oxidation of Met residues in proteins can lead to the destruction of protein structure and function. Methionine sulfoxide reductase 1 (MSRB1/SelR) can catalyze the reduction of methionine sulfoxide to Met, acting as a protective mechanism to inhibit protein oxidative damage (Labunskyy et al., 2014; Levine et al., 2000). Therefore, MSRB1 is considered an important antioxidant enzyme involved in protein repair and its expression is effectively regulated by Se content (Tarrago et al., 2022). In addition, RSL3 also has the ability to bind to other selenoproteins, including TXNRD1, selenoprotein K (SELENOK/SelK), and selenoprotein T (SELENOT/SelT), which further increases the possibility of other selenoproteins participating in the regulation of ferroptosis (Gao et al., 2018). When the body is in a Se-deficient state, the sensitivity of cells to ferroptosis increases, the antioxidant function of GPX4 and other selenoproteins against lipid peroxidation decreases, and the level of intracellular peroxidation products increases, causing cell damage and even ferroptosis, which is a landmark event of ferroptosis.

#### 3.1.4 Lipid homeostasis

The mechanism of ferroptosis is highly regulated by cellular lipid and its metabolism. Lipid metabolic disorders can cause nutritional iron deficiency in the body, disrupt iron homeostasis, and induce lipid peroxidation, accumulating LPO to directly damage cell membranes, leading to cellular dysfunction and ferroptosis (Ali-Rahmani et al., 2014; Chen et al., 2021b; Hadian and Stockwell, 2020; Stockwell et al., 2020). Fatty acids, cholesterol, and adipocyte metabolism abnormalities are the main causes of lipid homeostasis disorders (Kim J. W. et al., 2023). Studies (Bai et al., 2019; Chen et al., 2021c) have shown that the accumulation of free fatty acids in cells is promoted by lipid phagocytosis and the β-oxidation (FAO) of fatty acids, which mediates the release and metabolic abnormalities of fatty acids, inducing ferroptosis. Bayir et al. (2023) found that polyunsaturated fatty acid (PUFA)-containing LPO and peroxyl radicals can affect the fluidity, integrity, and stability of cell and organelle membranes, and are the “execution molecules” of ferroptosis. These special phospholipids are mainly PE containing AA or AdA, which can be converted to lipid peroxyl radicals (LPR, such as PE-AA-OO- and PE-AdA-OO-) through autoxidation, enzymatic catalysis, and the action of Fe^2^^+^ (Kagan et al., 2017). LPO oxidizes membrane phospholipids, causing changes in membrane fluidity and increased membrane permeability, and its degradation products, malondialdehyde (MDA) and 4-hydroxy-2-nonenal (4-HNE), can destroy embedded proteins and other biomolecules in the double-layer phospholipid membrane, ultimately leading to ferroptosis (Ayala et al., 2014; Stockwell et al., 2017). Acyl-CoA synthetase long-chain family member 4 (ACSL4) (Doll et al., 2017; Hassannia et al., 2021), lysophosphatidylcholine acyltransferase 3 (LPCAT3) (Reed et al., 2022), and arachidonate 15-lipoxygenase (ALOX15) (Ma et al., 2022) are key enzymes that participate in the biosynthesis and reconstruction of PE, activate PUFA, and affect its transmembrane characteristics, playing a key role in activating ferroptotic lipid peroxidation (Chen L. et al., 2024; Latunde-Dada, 2017).

Cholesterol is crucial for maintaining cell membrane integrity, fluidity, and microscopic structure (Forcina and Dixon, 2019). The mevalonate pathway (MVA) is the main pathway for cholesterol synthesis and is one of the important metabolic pathways in cells (Juarez and Fruman, 2021). MVA affects ferroptosis in three different ways, including GPX4, squalene (SQS), and coenzyme Q10 (CoQ10). A study (Kitsugi et al., 2023) has shown that blocking the rate-limiting enzyme of the MVA pathway can damage GPX4 dependence, making cells sensitive to ferroptosis. Freitas et al. (2024) observed that 7-dehydrocholesterol (7-DHC) can reduce the formation of lipid oxidation and truncated phospholipids, helping cells resist ferroptosis, and may be a potential regulator of lipid peroxidation and ferroptosis. This mechanism may bring new molecular targets and treatment strategies for diseases with related mutations. In addition, SQS downstream of IPP is involved in cholesterol synthesis, and CoQ10 is also downstream of IPP and participates in maintaining mitochondrial respiratory function. Inhibiting SQS activity can block ferroptosis, while inhibiting CoQ10 production can lead to mitochondrial respiratory dysfunction and oxidative damage, thereby promoting the progression of ferroptosis (Hassannia et al., 2019). Fe^2^^+^ can induce the expression of lipid and cholesterol metabolism enzymes such as low-density lipoprotein receptor (LDL-R) and fatty acid desaturase 1 (FADS1) in adipocytes, produce ROS through Fenton reactions, increase membrane-bound cholesterol, and reduce membrane fluidity (Bernotti et al., 2003; Drynda et al., 2010; Liu et al., 2013; Suwalsky et al., 2005).

Adipocytes also have a close relationship with ferroptosis. Adipocyte-enhanced protein 1 (AEBP1) is a transcriptional repressor involved in the regulation of multiple key biological processes, such as adipogenesis and inflammation (Majdalawieh et al., 2020). The iron level in adipocytes can also regulate the transcription and serum protein levels of adiponectin (Gabrielsen et al., 2012). These findings may provide new ideas and targets for the treatment of ferroptosis-related diseases. In addition, iron metabolism can regulate lipid metabolism by acting on lipid metabolic targets to achieve the regulation of OS occurrence (Table 1). Its existence can also increase the occurrence of OS in the body, damage DNA, proteins, and lipids, and further exacerbate lipid metabolic disorders (Jomova and Valko, 2011). A study (Galluzzi et al., 2012) has shown that cells with PUFA-containing cell membranes are more likely to undergo peroxidation reactions in the presence of Fe^2^^+^, and this reaction speed is greatly increased.

**Table 1:** Iron metabolism directly regulates lipid metabolism.

**Target**	**Function**	**Indications**
SREBP-2 (Zhang J. J. et al., 2017)	Controls cholesterol and fatty acid biosynthesis	Hypercholesterolemia
TNF-α (Fisher et al., 2021; Pang et al., 1996)	Inflammation-mediated lipid deposition	Hypercholesterolemia, inflammation, atherosclerosis
Adiponectin (Becker et al., 2015; Munoz Alferez et al., 2019; Wlazlo et al., 2013)	Activity of lipoprotein lipase and hepatic lipase	Obesity, diabetes, inflammation, atherosclerosis
Mitochondria (Cavaliere et al., 2023; Jadhav et al., 2021)	Mitochondrial function	Obesity and hepatic steatosis
Fe^2^^+^, HDL-C, total cholesterol (Dabbagh et al., 1997; Schonfeld et al., 2007)	Fatty acid oxidation and lipid transport	OS reaction
FXR (Tschuck et al., 2023)	Synthesis and excretion of bile acids	Hyperfat and hypercholesterolemia

#### 3.1.5 Amino acid homeostasis

Amino acid metabolism provides the body with proteins, energy substrates, GSH, and neurotransmitters, and regulates multiple lipid antioxidant systems to control ferroptosis. Among them, the relationship between cysteine (Cys) metabolism and ferroptosis is the closest. The body obtains Cys through diet, Met transulfuration, and GSH decomposition, and then synthesizes GSH, coenzyme A (CoA), GPX4, iron-sulfur clusters, and hydrogen sulfide (H_2_S) to inhibit ferroptosis (Fujii et al., 2020). Ferroptosis-inducing erastin targets a membrane amino acid transporter, the Xc-system (Dolma et al., 2003). Intracellular Cys mainly comes from the reduction of extracellular CySS taken up by the Xc-system, and the SLC7A11 is responsible for the uptake of extracellular CySS (Koppula et al., 2017; Soria et al., 2016). Cys is the rate-limiting substrate for GSH synthesis and also participates in the formation of Sec, which is involved in the formation of the GPX4 catalytic unit (Zhang Y. et al., 2021). GSH is a water-soluble tripeptide containing a γ-acyl amide bond and a thiol group, synthesized from Glu, Cys, and glycine(Gly), retaining the reducing thiol group of Cys, which gives GSH certain reducing properties and plays an important role in antioxidant defense (Kennedy et al., 2020; Lin et al., 2024). GSH can form a Fe-S complex with Fe^2^^+^, bind to the PCBP family to form the PCBP1-Fe-GSH complex, control the redox activity of the LIP in the cytoplasm, and inhibit ferroptosis (Bayir et al., 2020; Patel et al., 2019). GSH also reacts with GPX4 in an oxidation-reduction reaction, reducing H_2_O_2_ produced by SOD to H_2_O, and reducing lipid hydroperoxides (lipid-OOH) to non-toxic lipid alcohols (lipid-OH), reducing the accumulation of intracellular lipid-OOH (Kuang et al., 2020). In addition, the thiol group of GSH can act as a hydrogen donor, catalyzing the reduction of deoxyribonucleic acid to DNA, promoting DNA synthesis, and playing a crucial role in maintaining the expression and repair state of cell nuclear DNA (Morris et al., 2014). The reduction of GSH levels inhibits GPX4 function, leading to the accumulation of toxic peroxides, protein and cell membrane damage, and subsequent ferroptosis. Therefore, the Cys-GSH-GPX4 pathway plays an important role in the regulation of ferroptosis.

Nicotinamide adenine dinucleotide (NADH/NAD^+^) and its phosphorylated forms (NADPH/NADP^+^) are also important cofactors and core metabolites in the body and are closely related to the synthesis of amino acids, often used as key indicators to evaluate cellular metabolism (Christensen et al., 2017; Jeon et al., 2012; Ju et al., 2020). NADPH has multiple functions such as maintaining the reducing state of GSH, protecting membrane proteins, hemoglobin, and preventing the oxidation of enzyme protein thiol groups, and the levels of NADPH in the cytoplasm and cytosol have regulatory effects on ferroptosis (Ding et al., 2020; Doll et al., 2019; Glatzle et al., 1968). Glutathione reductase (GR) can use NADPH to catalyze the reduction of oxidized glutathione (GSSG) to GSH, eliminating LPO on the cell membrane and preventing cell membrane damage, thereby inhibiting ferroptosis (Yang et al., 2014). Therefore, NADPH can promote the production of a series of antioxidant products, thereby inhibiting ferroptosis. However, recent studies have found that NADPH can also promote the occurrence of ferroptosis. The PPP pathway in the cytoplasm is one of the main ways to produce NADPH, with the main rate-limiting enzymes being glucose-6-phosphate dehydrogenase (G6PDH) and 6-phosphogluconate dehydrogenase (6PGDH). A study (Dixon et al., 2012) has found that using shRNA to knock down G6PD or 6PGD in cells or using specific inhibitors of the PPP pathway, such as 6-aminonicotinamide (6-AN), can significantly inhibit erastin-induced ferroptosis, suggesting that NADPH produced by the PPP pathway may promote ferroptosis. The dual regulatory effect of NADPH on ferroptosis may be due to the different subcellular distributions of the enzymes in each pathway and the different products generated, but the antioxidant effect of maintaining cellular redox balance may exceed its potential pro-ferroptotic function (Zheng and Conrad, 2020).

Experiments (Badgley et al., 2020) have shown that inhibiting intracellular Cys metabolism and regulating the synthesis pathways of GSH and CoA by inhibiting panthothenate kinase (PANK) can effectively promote ferroptosis. CoA is a precursor for the synthesis of CoQ10, and CoQ10 and ferroptosis inhibitor protein 1 (FSP1) are another class of LPO reduction systems parallel to GSH/GPX4 (Bersuker et al., 2019; Doll et al., 2019). A study (Leu et al., 2019) found that CoA inhibits Glu overloading, erastin, or Cys deprivation-induced ferroptosis in multiple cell lines. FSP1, as an oxidoreductase, can reduce CoQ10 on the cell membrane, produce lipophilic free radical scavenging antioxidants, and prevent the increase of LPO (Bersuker et al., 2019). Therefore, regulating Cys metabolism to affect the levels of CoA, CoQ10, and FSP1 is an important pathway for cells to reduce LPO and inhibit ferroptosis. In addition, Cys can also regulate ferroptosis by regulating the metabolism of Fe^2^^+^ and H_2_S (Alvarez et al., 2017; Poltorack and Dixon, 2022). Since NA(D)PH, GSH, GPX4, CO-A, Fe2+, and H2S mediate the reduction of LPO and the production of ROS to regulate iron death, Cys is an important factor in the regulation of cellular iron death. In addition to Cys, there are many amino acid metabolism involved in the regulation of iron death. For example, the sulfur transfer synthesis of Met is the main way to obtain Cys in the liver during the synthesis of GSH, and H2S can also be generated through its catabolism to regulate iron death (Sanderson et al., 2019; Zhang T. et al., 2020). The MVA pathway is involved in the synthesis of Sec and CoQ10, which are catalytic centers of GPX4 and reducing agents to inhibit lipid peroxidation, respectively. In conclusion, the metabolism of many amino acids (including Cys, Met, etc.) is involved in the regulation of iron death, which may be a new target for the treatment of iron death-related diseases.

### 3.2 Other biological regulatory mechanisms of ferroptosis

#### 3.2.1 Autophagy

**Figure 3:**
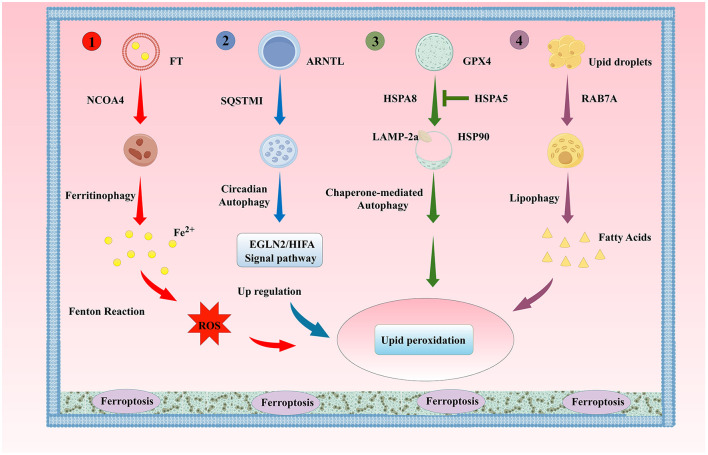
The Classic Autophagy Regulatory Mechanism of Ferroptosis. (1). Ferritinophagy; (2). Lipophagy; (3). Clock Autophagy; (4). CMA ARNTL, aryl hydrocarbon receptor nuclear translocator-like protein; SQSTM1/P62, sequestosome 1; HSPA8, heat shock protein family A member 8; HSPA5, heat shock protein family A member 5; HSP90, heat shock protein 90; LAMP-2a, lysosomal-associated membrane protein 2A; RAB7A, Ras-related protein Rab-7a.

Autophagy is a physiological process dependent on lysosomes, which degrades damaged macromolecules or organelles in cells to maintain intracellular homeostasis, including macroautophagy, microautophagy, and molecular chaperone autophagy (CMA) (Galluzzi and Green, 2019). In addition to these common types of autophagy, there are also selective autophagy forms with specific substrate degradation, including mitochondrial autophagy, lipid droplet autophagy, aggregate autophagy, endoplasmic reticulum autophagy, and ribosomal autophagy (Ma et al., 2024). Autophagy at different levels can enhance the ability of cells to survive under stress or induce autophagic cell death. Ferroptosis is a programmed cell death dependent on intracellular iron ion concentration and lipid peroxidation. Although the two are different physiological regulatory methods for cells facing various stresses, increasing evidence shows that autophagy and ferroptosis are closely related, and certain selective autophagy forms can regulate ferroptosis through modulating intracellular iron storage and oxidative stress levels. For example, the excessive activation of ferritinophagy, lipophagy, clock autophagy, and CMA can promote ferroptosis by degrading iron proteins, lipid droplets, circadian rhythm regulators, and GPX4, respectively (Honma et al., 2023; Juste et al., 2021) (Figure 3). Dysfunction of organelles such as mitochondria and endoplasmic reticulum is also an object of autophagy regulation of cellular ferroptosis. For example, mitochondrial autophagy can maintain a healthy number of mitochondria, prevent damage caused by mitochondrial morphology destruction, reduced oxidative phosphorylation activity, mitochondrial membrane potential collapse, and ATP synthesis, enhance mitochondrial autophagy, inhibit mitochondrial metabolic processes, reduce GSH rapid consumption caused by Cys deprivation, lipid ROS generation, and ferroptosis (Gao et al., 2019; Li Y. et al., 2021). Autophagy regulatory factors such as BECN1, human cathepsin B (CTSB), high mobility group protein B1 (HMGB1), phosphatidylethanolamine-binding protein 1 (PEBP1), mTOR, AMPK, and dual-specificity phosphatase 1 (DUSP1) also affect the ferroptosis process under certain conditions. BECN1, when phosphorylated by p38 mitogen-activated protein kinase (p38MAPK), can directly bind to the subunit SLC7A11 of the Xc- system, blocking the ability of the Xc- system to transport Cys, inhibiting GSH synthesis, and promoting ferroptosis (Kang et al., 2018; Song X. et al., 2018). ROS are direct inducers of ferroptosis and can also induce autophagy. In the ferroptosis induced by erastin dependent on autophagy, the molecular mechanism of ROS mediating the induction of autophagy and ferroptosis, as well as the synergistic biological effects produced by the two, are important directions for future research and discussion (Gao et al., 2016; Hou et al., 2016). Autophagy and ferroptosis interact and play key roles in the occurrence and development of neurodegenerative diseases such as AD.

#### 3.2.2 Endoplasmic reticulum stress

**Figure 4:**
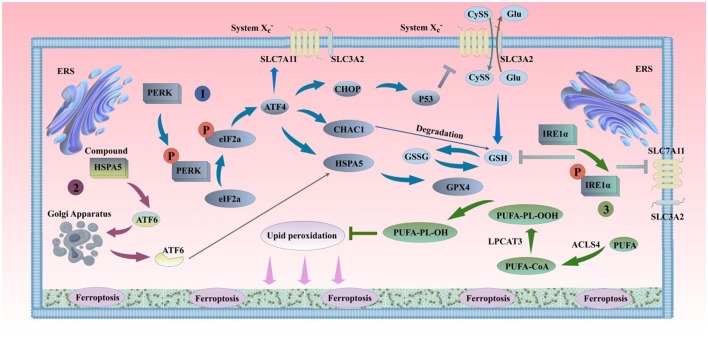
ERS and ferroptosis. (1). PERK can oligomerize and undergo trans-autophosphorylation under ERS conditions, inhibiting protein translation by phosphorylating eIF2α, thereby reducing the entry of proteins into the overloaded ER. The phosphorylation of eIF2α by PERK selectively translates the mRNA of ATF4, alleviating ERS. The activation of the PERK pathway in the short term can protect cells by inhibiting protein synthesis and reducing unfolded proteins in the ER. However, the long-term activation of the PERK pathway can damage cell viability. The sustained activation of the PERK pathway can induce the accumulation of CHOP and the expression of CHAC1, inhibiting SLC7A11 and GSH, and promoting the occurrence of ferroptosis. On the other hand, ATF4-mediated HSPA5 expression prevents the degradation of GPX4, inhibiting ferroptosis. (2). Under ERS conditions, ATF6 is released from the HSPA5 complex and transferred to the Golgi apparatus in vesicles. ATF6 is sequentially cleaved by S1P and S2P, releasing transcriptionally active ATF6. Active ATF6 leads to increased HSPA5 expression, which promotes the correct folding and transport of unfolded or misfolded proteins and further affects the sensitivity of cells to ferroptosis by the binding of HSPA5 to GPX4, thereby alleviating ERS and maintaining the normal function of the ER. (3). When unfolded proteins accumulate in the ER, IRE1 undergoes dimerization and trans-autophosphorylation, promoting ER protein folding, secretion, and activation of phospholipid biosynthesis and ER-associated degradation pathways. If ERS continues or worsens, IRE1α is further activated. The mRNA transcripts of GCL subunits (GCL) and SLC7A11 are newly identified targets of IRE1α negative regulation (Jiang et al., 2024). IRE1α determines the sensitivity of cells to RSL3 and ferroptosis by negatively regulating SLC7A11 and GCL. PERK, protein kinase RNA-like endoplasmic reticulum kinase; eIF2α, eukaryotic translation initiation factor; ATF4, transcription factor 4; CHOP, C/EBP homologous protein; S1P, site-1 protease; S2P, site-2 protease; CHAC1, glutathione-specific gamma-glutamylcyclotransferase 1; P53, cellular tumor antigen p53; ATF6, transcription factor 6; IRE1α, inositol-requiring kinase 1α.

The endoplasmic reticulum (ER) is highly sensitive to stress affecting intracellular energy levels, oxidative states, or calcium ion concentrations. When cells are exposed to external stimuli such as hypoxia and drug toxicity, the oxidative environment of the ER is disrupted, leading to ER dysfunction and the accumulation of unfolded or misfolded proteins in the ER lumen, a state known as ER stress (ERS) (Wu Z. et al., 2018). Once the homeostasis of the ER is disrupted, a series of cascading reactions are activated, which are closely related to the occurrence of ferroptosis (Figure 4). The main types of ERS-activated signaling pathways are: the unfolded protein response (UPR); ER overload response (EOR); sterol regulatory cascade (Huang et al., 2024). Both UPR and EOR are caused by protein processing disorders, while the sterol regulatory cascade is triggered by the loss of cholesterol synthesized on the ER surface. UPR is a self-protection mechanism of cells against ERS, helping to maintain intracellular homeostasis (Walter and Ron, 2011). However, in the aging process, persistent ERS and chronic inflammation can also activate UPR, directly leading to the accumulation of damage in the body and the exacerbation of complications. EOR refers to the overaccumulation of correctly folded proteins in the ER, leading to the activation of a series of signaling substances. EOR can activate the nuclear transcription factor NF-κB, inducing the production of inflammatory proteins, interferons, and interleukins such as IL-1β, IL-6, and IL-12, and ultimately initiating cell survival, apoptosis, inflammation, and differentiation-related signaling pathways (Ghosh et al., 2012; So, 2018). In addition, EOR can be inhibited by antioxidants and calcium channel antagonists, and can be activated by drugs that promote the release of calcium ions, indicating that EOR may be related to calcium storage and ROS production (Lin et al., 2009). ROS accumulation can not only activate ERS responses but also lead to increased intracellular LPO, ultimately promoting cell ferroptosis. Caspase-12, a member of the cysteine protease family, is mainly located in the ER. ERS causes the activation of caspase-12, which in turn activates other caspase family members, acting as a key medium for mediating programmed cell death (Fischer et al., 2002). ERS activates a series of cascading reaction pathways, regulating the formation of intracellular iron ions and ROS through iron metabolism, Xc-/GPX4 system (Zhao et al., 2021), lipid metabolism (Tak et al., 2022), and other ferroptosis pathways such as p62-Keap1-NRF2, p53-SAT1-ALOX15, ATG5-ATG7-NCOA4, and glutaminolysis (Lane et al., 2018), playing a dual role in ferroptosis.

#### 3.2.3 Epigenetics

Epigenetics, an emerging genetic theory in the 21^st^ century, refers to the regulatory process of gene expression without changing the DNA sequence through the addition of chemical modifications (Avci et al., 2022). The main mechanisms of epigenetics include DNA methylation, histone modification, chromatin remodeling, and non-coding RNA transcription, which have been proven to be closely related to important physiological processes such as gene transcription, cell fate determination, growth and development, and immune microenvironment (Li, 2021). Lymphocyte-specific helicase (LSH), histone demethylase (KDM3B), Se, and non-coding RNA (ncRNA) have all been reported to participate in the epigenetic regulation of ferroptosis. LSH is an ATP-dependent helicase involved in the development and metabolism of normal cells. LSH can change the methylation status of histones, upregulate the expression of SCD1 and FADS2, reduce ROS produced in lipid metabolism and intracellular iron accumulation, and increase the synthesis of CoQ10 to inhibit ferroptosis (Jiang et al., 2017; Tesfay et al., 2019). Regarding KDM3B, Wang Y. et al. (2020) reported its involvement in erastin-induced ferroptosis, but the specific regulatory upstream and downstream relationships are still unclear. Therefore, more experiments are needed to explore the mechanism by which KDM3B regulates ferroptosis.

In addition, ncRNA also plays an important role in the epigenetic regulation of ferroptosis. MicroRNAs (miRNAs) mainly negatively regulate the expression of target genes by targeting the3′UTR region. Currently, miRNAs have been proven to affect the body's redox balance by regulating CySS metabolism (Peng et al., 2024; Wu et al., 2017a), lipid metabolism (Busch et al., 2015; Wu et al., 2017b), and iron metabolism pathways (Andolfo et al., 2010; Ripa et al., 2017) in various ways. Long non-coding RNAs (lncRNAs) have also been proven to be closely related to the occurrence of ferroptosis. lncRNA-ZFAS1 can reduce the degradation of SLC38A1 by competitively binding to miR-150-5p, thereby promoting lipid synthesis substrate Glu transport and promoting pulmonary fibrosis and ferroptosis (Yang et al., 2020). IncRNA-PVT1 can inhibit ferroptosis in atherosclerotic damage by adsorbing miRNAs as a sponge, reducing the binding of miR-106a to CCND1 (Lu et al., 2020). Although research on ferroptosis has deepened in recent years, the exploration of the epigenetic mechanisms regulating ferroptosis is still insufficient, and there are relatively few reference literatures. In the future, the field of ferroptosis epigenetics should be further expanded.

## 4 Relationship between Alzheimer's disease pathogenesis and ferroptosis

In 1953, increased iron levels were first detected in the brains of AD patients (Goodman, 1953). More and more evidence shows that iron metabolism disorder is closely related to AD pathology, and iron and ferritin deposition can exist in SP, NFT and blood vessels in AD brain (Smith et al., 1997). Smith et al. (2010) found that iron content increased in the early stage of AD, that is, mild cognitive impairment (MCI), and abnormal iron metabolism could promote the neurodegeneration of AD. Iron deposition in the basal ganglia and reduction in blood perfusion in multiple regions was observed during the progression of MCI to AD (Li D. et al., 2020). Antharam et al. (2012) used MRI diagnostic technology to find that the accumulation of Aβ in the early stage of AD is accompanied by an increase in iron concentration, and brain iron deposition may directly affect the formation of AD pathological symptoms. Based on SWI technology, Haller et al. (2021) found pathological iron deposition in the hippocampus and substantia nigra of AD patients, which was highly correlated with the progression of the disease. Yao et al. (2024) studied brain slices from AD patients and found increased paramagnetic and diamagnetic susceptibility in the medial prefrontal, medial parietal, and hippocampal paracortex regions of the brain, which was related to iron deposition and Aβ accumulation in AD. This was also verified in APP/PS1 double transgenic mice. Allison McIntosh et al. (2019) found iron deposition in the hippocampus of APP/PS1 mice using MRI technology, and histological analysis showed that small glial cells with Aβ deposition in the brain had increased iron overload. Notably, there are differences in iron content in different brain regions of AD patients. Compared with the normal control group, the iron content in the left frontal lobe, parietal lobe, temporal lobe, occipital lobe, and parietal cortex of AD patients increased, with some sparse clusters of iron distributed in the right hemisphere, which could be co-localized with Aβ plaques (Yang A. et al., 2022). The early increase in iron observed in the cortex of PSAPP mice begins at 24 weeks of age, whereas hippocampal iron content rises from 13 to 24 weeks of age, followed by a subsequent decline in hippocampal iron levels between 24 and 56 weeks of age (Li D. et al., 2020). Notably, in contrast to the fluctuating iron dynamics observed in the hippocampus, the pronounced and sustained iron accumulation in the cerebral cortex establishes this region as a highly promising focus for clinical diagnostic applications.

**Figure 5:**
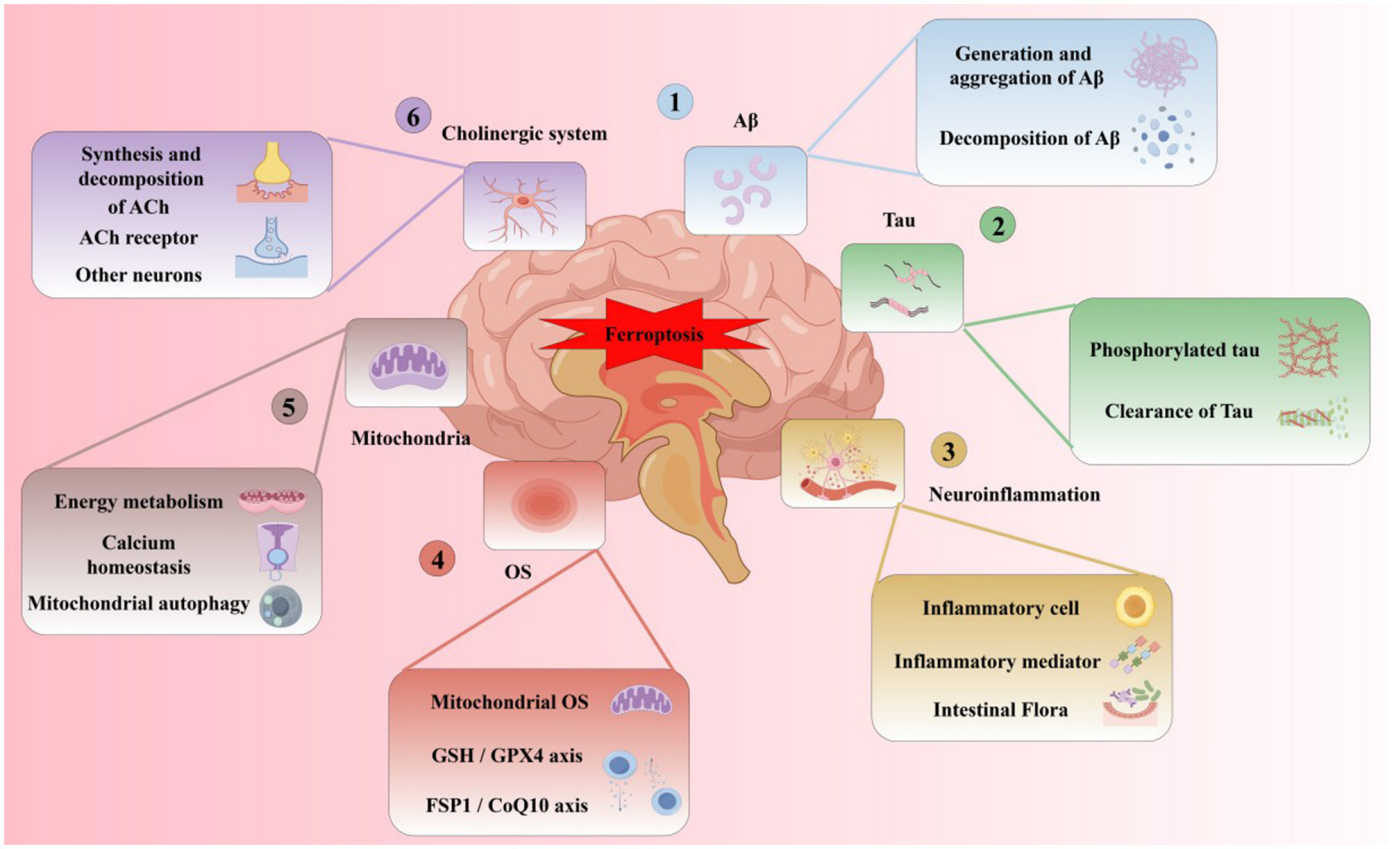
Six major pathogenesis of ferroptosis and AD.

Subsequent research (Yan and Zhang, 2019) found that in the brains of AD patients, the imbalance of iron metabolism-related proteins may lead to the disturbance of iron metabolism in neurons, resulting in greater net iron inflow than net outflow, inducing iron overload, and forming iron deposition in related brain areas. The specific classification, function and changes of iron metabolism-related proteins in AD brain are shown in Table 2. In addition, with the deepening of research, the hypothesis of the pathogenesis of AD has been gradually enriched. A recent review on the pathogenesis of AD (Zhao et al., 2022) shows that Aβ plaque aggregation, NFT induced by Tau lesions, neuroinflammation and mitochondrial dysfunction are the six major hypotheses for the pathogenesis of AD at present. At present, from basic to clinical and combined with internal and external experiments, the multi-angle and multi-level studies suggest that AD has abnormal iron metabolism. Iron death may be an important step to participate in the course of AD and is closely related to the six major pathogenesis (Figure 5).

**Table 2:** Classification and function of iron metabolism-related proteins and their changes in AD brain.

**Protein type**	**Protein name**	**Function in iron metabolism**	**Changes in AD brain**
Iron regulatory protein	Hepcidin (Hepc) (Li et al., 2011)	Degradation of FPN, inhibition of cellular iron efflux, maintenance of iron homeostasis	Hepc levels rise, but its impact on AD depends on the level of inflammatory factors in the brain
Iron storage protein	FT (Vidal et al., 2008; Zhang N. et al., 2021)	Storage of intracellular iron	FTH1, FTL, and mitochondrial ferritin (MtFt) all increase, with FTL positively correlated with the formation of senile plaques and neuronal death
Iron transport protein	FPN1 (Bao et al., 2021)	Efflux of cellular Fe^2^^+^	FPN1 levels decrease
	DMT1 (Zheng et al., 2009)	Uptake of cellular Fe^2^^+^	DMT1 levels increase
	TF (Hoshi et al., 2021)	Transport of Fe3^+^ in the body	TF levels increase
	TFR1 (Kang et al., 2024)	Uptake of Fe3^+^	TFR1 levels increase
Iron oxidation protein	Ceruloplasmin (CP) (Zanardi and Alessio, 2021)	Ferrous oxidase activity, assistance in iron efflux	Active CP levels decrease in serum, inactive apocp levels increase, overall CP activity decreases
	Calprotectin/ S100A8/A9 (Tian et al., 2024)	Fe3^+^ reductive activity, coordination with Fe^2^^+^	S100A8/A9 significantly increase in microglia and Aβ deposits, promoting neuroinflammation

### 4.1 Relationship between Aβ and ferroptosis in Alzheimer's disease

#### 4.1.1 Relationship between abnormal Aβ production/aggregation and ferroptosis in AD

**Figure 6:**
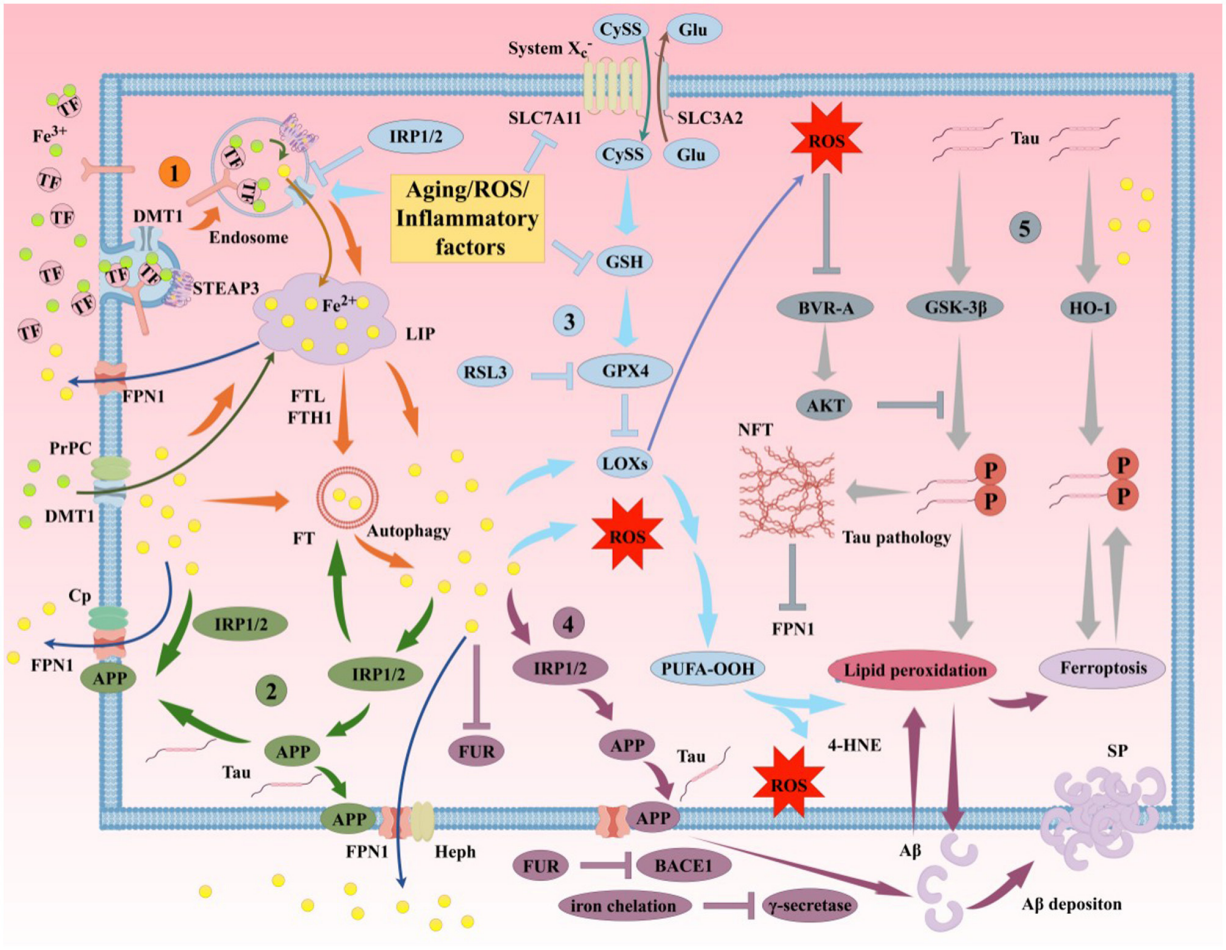
Iron participates in the formation of AD's primary pathological mechanisms through multiple pathways. The aggregation of Aβ plaques and hyperphosphorylation of Tau protein are closely associated with ferroptosis. (1). Mechanisms of iron uptake in AD. Neurons uptake iron via the TF/TFR1 complex or through DMT1/PrPC-dependent pathways. TF undergoes autophagy mediated by NCOA4, releasing iron and leading to lethal iron levels and ferroptosis. (2). Mechanisms of iron efflux in AD. FPN1/Cp or FPN1/Heph facilitates iron efflux with the assistance of APP, which stabilizes FPN1 via soluble Tau transport. Aging, inflammation, and OS dysregulate iron transporters, causing iron retention. (3). Role of the GSH/GPX4 pathway in AD ferroptosis. Under certain conditions, reduced GPX4 or GSH in neurons fails to counteract lipid peroxidation. Accumulation of PUFA-OOH to lethal levels via Fenton reactions or ALOX catalysis induces Tau hyperphosphorylation, Aβ formation, and neuronal loss. (4). Molecular mechanisms of Aβ plaque formation and aggregation. Iron overload upregulates FT, FPN1, and APP expression via IRP-IRE interactions while inhibiting normal FUR function, leading to BACE1 upregulation and increased extracellular Aβ deposition and lipid peroxidation. (5). Molecular mechanisms of Tau hyperphosphorylation. Iron promotes Tau hyperphosphorylation, aggregation, NFT formation, and lipid peroxidation. Overexpression of GSK-3β and HO-1 induces Tau phosphorylation, leading to cerebral accumulation and NFT formation. Elevated GPX4 expression inhibits this process. Reduced soluble Tau increases brain iron deposition by suppressing FPN1 activity, exacerbating ferroptosis. Aβ, β-amyloid; Tau, microtubule-associated protein; APP, amyloid precursor protein; PrPC, prion protein; Cp, ceruloplasmin; Heph, hephaestin; FUR, furin protease; IRP, iron regulatory protein; BACE1, β-secretase 1; HO-1, heme oxygenase-1; SP, senile plaques; NFT, neurofibrillary tangles.

The aggregation of Aβ plaques and hyperphosphorylation of Tau protein, as the primary pathological features of AD, are closely related to ferroptosis (Figure 6). Aβ is generated through sequential cleavage of amyloid precursor protein (APP) by β-secretase 1 (BACE1) and γ-secretase (Guillemot et al., 2013). Elevated iron levels in the brain promote APP expression and subsequent progression of the amyloidogenic process. Intracellular iron regulates APP translation by targeting the iron-responsive element (IRE) in the5′ untranslated region (UTR) of APP mRNA. When iron binds to iron regulatory proteins (IRPs), the inhibition of APP mRNA is weakened, leading to increased APP translation and enhanced Aβ production in the brain (Rogers et al., 2002). Under high iron conditions, the concentration of furin protease (FUR) decreases, thereby enhancing BACE1 activity, promoting APP cleavage, and increasing Aβ levels (He et al., 2023; Silvestri and Camaschella, 2008). Iron chelators can reduce BACE1 and γ-secretase activity, inhibiting Aβ production (Mandel et al., 2008). Iron deposition not only increases Aβ generation in the brain but also promotes Aβ deposition and oxidative stress (OS)-induced toxicity (Lane et al., 2018; Li et al., 2013). Additionally, APP has been identified as an anchoring protein for ferroportin 1 (FPN1), stabilizing FPN1 expression on the cell surface to promote iron efflux (Li et al., 2019). In mouse models, APP depletion leads to intracellular iron accumulation, while exogenous APP supplementation or overexpression rescues this phenomenon (Wan et al., 2012). APP regulates intracellular iron metabolism homeostasis by stabilizing FPN1 on the cell surface, thereby enhancing iron efflux. Thus, brain iron modulates Aβ production by regulating APP expression and BACE1/γ-secretase activity.

Beyond iron metabolism, Se homeostasis during ferroptosis is also linked to AD. Se and selenoproteins inhibit Aβ aggregation, significantly reducing Aβ-mediated cytotoxicity and blocking the AD cascade. Song G. L. et al. (2018) demonstrated that Se intervention in AD model mice downregulated BACE1 levels and markedly reduced Aβ production in the brain. Zhou et al. (2021) found that rats injected with Aβ and treated with selenium quantum dots exhibited shorter escape latencies in water maze tests compared to pre-intervention groups. A study (Hambright et al., 2017) showed that GPX4-knockout AD mouse models exhibited reduced hippocampal and cortical neuronal proteins and increased LPO, which were reversed by the lipophilic antioxidant vitamin E. This indicates that GPX4 ameliorates ferroptosis-induced neurodegeneration and cognitive impairment, alleviating synaptic loss and neuronal dysfunction in AD. In addition to GPX4, selenoproteins such as SELENOR and SELENOP may protect APP from the negative effects of iron deposition (Aaseth et al., 2016; Wang et al., 2017). NADPH oxidase also lowers pH, potentially increasing BACE1 activity and promoting Aβ generation in the brain (Salminen et al., 2013). Furthermore, studies (Kang et al., 2018; Song X. et al., 2018) revealed that AMPK-mediated phosphorylation of BECN1 at Ser 90/93/96 is essential for lipid peroxidation. Abnormal Aβ accumulation exacerbates ROS production and induces OS, further contributing to redox imbalance in the AD brain (Sharma and Kim, 2021).

#### 4.1.2 Relationship between abnormal Aβ clearance and ferroptosis in AD

Under physiological conditions, Aβ is cleared via receptor-mediated transport across the BBB to maintain its physiological levels. A study (Abad-Rodriguez et al., 2004) shows that mild reductions in membrane cholesterol promote BACE1-mediated APP cleavage, increasing Aβ production and SP formation. Conversely, elevated membrane cholesterol reduces insulin-degrading enzyme (IDE) and neprilysin (NEP) activity, impairing Aβ transport and clearance, thereby promoting SP formation (Wang et al., 2019). Cholesterol also interacts closely with sphingolipids, forming lipid rafts that anchor BACE1 and the γ-secretase complex (Wang et al., 2019). In early AD stages, lipid raft composition in the frontal and entorhinal cortices is altered, accompanied by BACE1 accumulation in lipid raft domains. Apolipoprotein E (APOE), the primary apolipoprotein and cholesterol transporter in the central nervous system (Chen et al., 2021d), drives lysosomal cholesterol sequestration in astrocytes, reducing lipid catabolism and cholesterol transport, leading to lipid dyshomeostasis and impaired Aβ clearance (Jeong et al., 2019). While the critical role of lipid metabolism and peroxidation in ferroptosis is established, the specific lipid peroxide species, generation mechanisms, subcellular localization, and cellular response pathways triggered by lipid peroxidation in AD remain poorly understood.

Autophagy dysfunction, associated with ferroptosis, has been confirmed in AD patients and animal models. Gu et al. (2022) identified nine hub genes linking iron metabolism to AD through differential analysis and weighted gene co-expression network analysis (WGCNA). Gene Ontology (GO) analysis revealed that these iron metabolism genes primarily participate in autophagy-related biological processes (e.g., ATP6V1G2, ATP6V1H, and ATP6V1D). ATP6V1G2 encodes the G2 subunit of vacuolar ATPase (V-ATPase), which transports protons from the cytoplasm to lysosomes and maintains lysosomal acidification (Li W. X. et al., 2020). V-ATPase deficiency disrupts autolysosomal function and suppresses autophagy (Williamson and Hiesinger, 2010), indicating a close link between iron metabolism and autophagy in AD. The autophagy inducer rapamycin exerts pharmacological effects by enhancing autophagy to clear intracellular Aβ aggregates, reduce extracellular SP burden, and improve cognitive function (Wu et al., 2022). This may involve shared regulators of ferroptosis and autophagy, such as mTOR and p38MAPK. Recent study (Liu et al., 2019) suggests that autophagy promotes ferroptosis by selectively degrading aryl hydrocarbon receptor nuclear translocator-like protein (ARNTL). Additionally, when intracellular iron levels decline, ferritin is degraded to release free iron—a process termed ferritinophagy. Biasiotto et al. (2016) proposed that abnormal ferritinophagy may link autophagy impairment and iron dyshomeostasis in AD. Research (Streit et al., 2022) indicates that ferroptotic droplet degeneration in the human brain leads to targeted Aβ deposition and SP formation. Further elucidation of the mechanisms linking ferroptosis to Aβ synthesis and degradation may provide broader prospects for AD treatment and prevention.

### 4.2 Relationship between tau protein and ferroptosis in AD

#### 4.2.1 Relationship between abnormal tau phosphorylation and ferroptosis in AD

Iron overload-induced lipid peroxidation promotes Tau polymerization, leading to hyperphosphorylation and NFT formation in the brain (Ayton et al., 2013; Gamblin et al., 2000). Studies (Guo et al., 2013; Jin Jung et al., 2013) show that iron overload activates the CDK5/p25 complex and glycogen synthase kinase-3β (GSK-3β), inducing Tau hyperphosphorylation in neurons. Vitamin D receptor (VDR) activation, lipid peroxidation inhibition, and elevated GPX4 expression may play key roles in suppressing this process (Chen L. L. et al., 2023). Iron deposition may also interfere with insulin signaling, causing Tau hyperphosphorylation (Wan et al., 2019). In humans, 90% of iron originates from hemoglobin degradation in senescent erythrocytes. Heme oxygenase-1 (HO-1), a rate-limiting enzyme in heme degradation, is critical for iron metabolism (Maamoun et al., 2019). In AD patients, HO-1 expression is significantly elevated in reactive astrocytes in the hippocampus and cerebral cortex (Schipper et al., 2009). Chronic HO-1 overexpression in AD mice induces Tau phosphorylation, cerebral accumulation, and NFT formation, exacerbating pathology (Hui et al., 2011). Astrocytic HO-1 activation serves as a potent transducer of harmful stimuli and may be a therapeutic target for AD ferroptosis. Similarly, hyperphosphorylated Tau, a pathological hallmark of AD, promotes cerebral iron deposition, creating a vicious cycle in AD progression (Wang F. et al., 2022). A study (Lei et al., 2012) shows that reduced soluble Tau in AD brains increases iron deposition by suppressing FPN1 activity. Tau deficiency may also disrupt iron release via APP expression regulation, leading to intracellular iron accumulation, iron deposition, and pathological Tau changes, further aggravating ferroptosis and neuronal damage (Duce et al., 2010; Tuo et al., 2017).

OS and ROS are also linked to Tau pathology. Superoxide reduction during OS accelerates peroxide synthesis, depleting intracellular GSH and NADPH reserves and reducing ROS resistance. High ROS levels interfere with Tau's affinity for microtubule-associated proteins (MAPs), causing microtubule network degeneration and subsequent Tau detachment and aggregation into NFTs (Haque et al., 2019; Wang S. et al., 2022). ROS also activate enzymes such as p38MAPK and GSK-3β to phosphorylate Tau, promoting NFT formation (Germann and Alam, 2020; Hugon and Paquet, 2021; Lin et al., 2020). HO-1 oxidizes cellular heme to produce biliverdin, free iron, and carbon monoxide (CO) (Perry et al., 2002). Released Fe^2^^+^ catalyzes Fenton reactions, generating high levels of endogenous ROS and perpetuating OS in brain cells (Bao et al., 2020; Ward et al., 2014; Zukor et al., 2009). Hydroxyl radicals from Fenton reactions attack proteins or biomolecules, forming Tau oligomers via Cys-Cys bonds or kinase pathways (Huang et al., 1999; Soeda et al., 2015; Uranga et al., 2009). Iron nanoparticles increase permeability in human microvascular endothelial cells, with ROS production and microtubule remodeling as key factors (Apopa et al., 2009). Elevated ROS enhances BBB endothelial permeability, raising intracellular iron levels and accelerating AD pathology (Wang J. et al., 2023). Iron chelators and antioxidants may serve as effective agents to inhibit ferroptosis-induced Tau aggregation.

Iron can also form intermolecular coordination complexes with phosphorylated amino acid residues to generate Tau oligomers (Bader et al., 2011; Nubling et al., 2012). Conversely, Se and selenoproteins reduce Tau phosphorylation and NFT formation. In AD mice, Se-Met normalizes synaptic proteins and reduces Tau phosphorylation via PP2A activation, rescuing cognitive deficits (Song G. et al., 2014). Se supplementation increases SELENOS expression, alleviates ER stress, and suppresses Tau hyperphosphorylation, delaying NFT formation (Rueli et al., 2017). SELENOP interacts with the C-terminal domain of α-tubulin, regulates microtubule assembly, and mitigates ROS burden by interacting with Tau, Ca^2^^+^, and polyamines, protecting microtubule structure and function (Yue et al., 2020). SELENOP plays a vital regulatory role in AD pathology through direct antioxidant effects and indirect roles as a Se donor for other selenoproteins. Inorganic Se compounds also influence AD progression. Studies (Corcoran et al., 2010; Jin et al., 2017) show that sodium selenate reduces Tau phosphorylation *in vivo* and *in vitro* by activating serine/threonine-specific protein phosphatase 2A (PP2A), rescuing cognitive deficits in Tau transgenic mice (e.g., P301L, K369I, and Tau441 models).

#### 4.2.2 Relationship between impaired tau clearance and ferroptosis in AD

Beyond Tau generation and modification, its clearance is also linked to ferroptosis. A study (Ando et al., 2020) found that reduced phosphatidylinositol-binding clathrin assembly protein (PICALM) exacerbates Tau pathology in AD mice. Genome-wide association studies (GWAS) identify PICALM as a major AD risk factor, second only to APOE and BIN1 (Van Acker et al., 2019). PICALM is widely expressed in neural tissues, particularly BBB endothelial cells (Kisler et al., 2023). Studies (Ando et al., 2013; Hattersley et al., 2021; Ravikumar et al., 2010) reveal abnormal cleavage of PICALM in AD brains, with significantly reduced 75 kDa PICALM levels, disrupted cathepsin D (Cath D) processing, and impaired lysosomal function, leading to autophagy deficits and inhibited Tau degradation. Hyperphosphorylated and aggregated Tau disrupts neuronal iron efflux, increasing iron in NFTs and perpetuating a vicious redox cycle (Wang and Mandelkow, 2016). Additionally, PICALM promotes clathrin-mediated endocytosis (CME) by facilitating clathrin-coated vesicle formation. Its expression restores CME function, transferrin receptor (TFR) expression, and intracellular iron levels (Dreyling et al., 1996; Scotland et al., 2012; Tebar et al., 1999; Yao et al., 2005). Free iron binds to metal transporters like DMT1 and TFR at the BBB, and BBB dysfunction exacerbates cerebral iron accumulation (McCarthy and Kosman, 2015; Zhao et al., 2023). Thus, PICALM dysfunction may further impair BBB integrity, promoting cerebral ferroptosis and inhibiting Tau degradation.

Astrocytes, key components of the BBB, play vital roles in suppressing ferroptosis and clearing Tau. A study (Fan et al., 2024) shows that lactoferrin (LF) overexpression in astrocytes reduces iron deposition in APP/PS1 mouse neurons, increases GPX4 expression, and inhibits ferroptosis-induced neuronal damage. Elevated iron deposition in AD brains correlates with increased expression of the AD risk gene APOE4 (Alim et al., 2019). Under physiological conditions, astrocytes rapidly internalize, process, and release Tau via exosomal mechanisms. APOE4 impairs astrocytic clearance of extracellular Tau, leading to pathogenic Tau accumulation (Eisenbaum et al., 2024). Narayan et al. (2020) found that APOE4 disrupts astrocytic endocytosis, which is rescued by PICALM overexpression. Tau clearance via the glymphatic system is another pathway. Impaired glymphatic function due to chronic hypoperfusion from BBB damage exacerbates cognitive deficits and Tau hyperphosphorylation (Wu et al., 2024). These findings suggest that PICALM dysfunction-induced BBB impairment is a key contributor to cerebral iron deposition and reduced Tau clearance.

CMA defects are also observed in AD. CMA activation via CA77.1 ameliorates pathological symptoms in AD mice with CMA key molecule knockdown, significantly reducing phosphorylated Tau (Bourdenx et al., 2021). However, a study (Fan et al., 2024) indicates that reducing astrocytic iron deposition and inhibiting CMA to enhance GPX4 expression effectively combat AD by suppressing ferroptosis. This suggests that CMA knockout and overactivation may differentially regulate ferroptosis, necessitating tight control of CMA expression. Postmortem studies (Hou et al., 2018, 2020) reveal elevated phosphorylated serine 65 ubiquitin (pS65-Ub), a marker of impaired mitophagy, in AD brains, correlating with early phosphorylated Tau deposition and mitochondrial autophagy dysfunction. Damaged mitochondria trigger OS, inducing Tau aggregation, which in turn hinders selective degradation of dysfunctional mitochondria (Kerr et al., 2017). Future research should explore signaling pathways and molecular mechanisms of autophagy-regulated Tau in ferroptosis, as well as dynamic changes across AD stages. Additionally, developing highly specific and effective Tau degradation modulators remains a critical direction.

### 4.3 Relationship between neuroinflammation and ferroptosis in AD

#### 4.3.1 Relationship between inflammatory cell abnormalities and ferroptosis in AD

**Figure 7:**
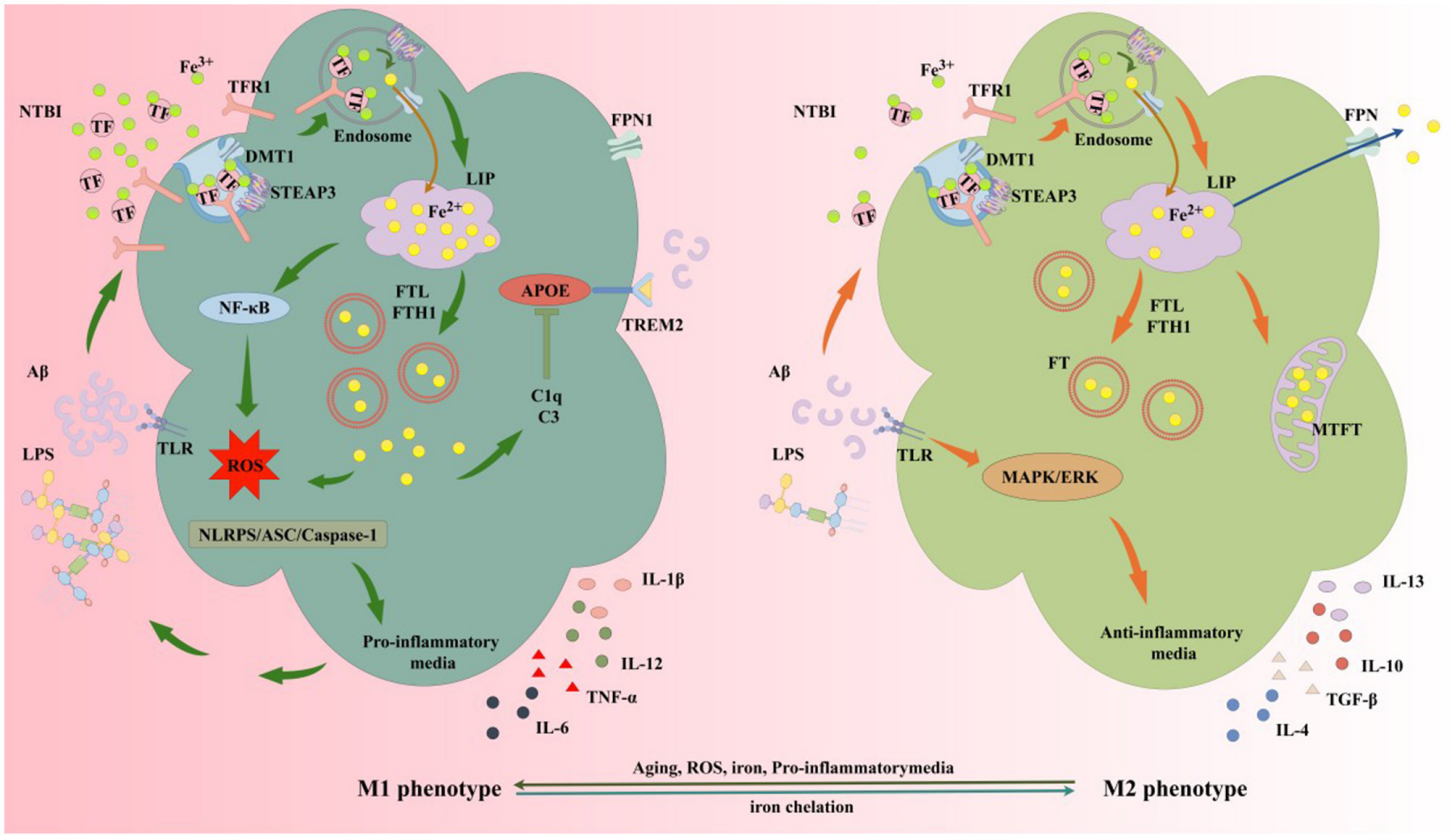
Iron and iron chelators modulate microglial M1/M2 polarization. The protective M2 microglial phenotype secretes anti-inflammatory mediators and neurotrophic factors, clearing or sequestering Aβ and Tau to protect neurons. M2 microglia upregulate DMT1 and FT expression, enhancing NTBI uptake, expanding iron storage in FT and MtFt, and compartmentalizing extracellular and intracellular iron. In AD, microglia exposed to elevated iron, LPS, and extracellular Aβ from damaged neurons favor M1 activation. High iron intake and impaired efflux increase the LIP in microglia, activating inflammatory pathways via iron-derived ROS, releasing cytokines, and exacerbating neuroinflammation. Elevated iron upregulates complement C3 and C1q while suppressing the APOE-TREM2 axis, reducing Aβ plaque phagocytosis, damaging neurons, and accelerating AD progression. MtFt, mitochondrial ferritin; LPS, lipopolysaccharide; TGF-β, transforming growth factor-β; TNF-α, tumor necrosis factor-α; IL-1β, interleukin-1β; IL-4, interleukin-4; IL-6, interleukin-6; IL-10, interleukin-10; IL-12, interleukin-12; IL-13, interleukin-13; TLR, toll-like receptor; MAPK, mitogen-activated protein kinase; ERK, extracellular signal-regulated kinase; C1q, complement 1q; C3, complement 3; NTBI, non-transferrin-bound iron; NF-κB, nuclear factor-kappa B; APOE, apolipoprotein E; TREM2, triggering receptor expressed on myeloid cells 2; ASC, apoptosis-associated speck-like protein; NLRP3, NOD-like receptor pyrin domain-containing 3.

Previous studies highlight neuroinflammation and microglial activation as key mechanisms in AD pathogenesis. Activated microglia exhibit dual roles across AD stages, including pro-inflammatory M1 and anti-inflammatory M2 phenotypes, with iron and iron chelators balancing M1/M2 polarization (Wang M. et al., 2023) (Figure 7). Iron accumulation induces glial activation via NF-κB-mediated inflammatory cytokine release (McCarthy et al., 2018; Nnah et al., 2020; Zhang et al., 2006). Activated microglia release inflammatory factors that impair astrocytic support for neuronal survival, growth, and synaptic homeostasis, worsening neurodegeneration (Alrouji et al., 2024; Kaur et al., 2019). Microglia-astrocyte crosstalk also amplifies inflammation. Astrocytes secrete factors like secreted frizzled-related protein 1 (SFRP1) that modulate microglial activation. In neuroinflammation, SFRP1 upregulates hypoxia-inducible factor (HIF)-dependent inflammatory pathways, promoting microglial activation (Rueda-Carrasco et al., 2021). Iron may exacerbate neurovascular uncoupling via astrocytic dysfunction and inflammation (Salami et al., 2021). Tumor necrosis factor-α (TNF-α) enhances iron uptake and retention in astrocytes and microglia by increasing DMT1 expression, while transforming growth factor-β1 (TGF-β1) promotes astrocytic iron efflux via FPN1 upregulation (Rathore et al., 2012). Pro-inflammatory cytokines may regulate cerebral iron homeostasis, creating a positive feedback loop that aggravates neuroinflammation in AD.

#### 4.3.2 Relationship between inflammatory mediator abnormalities and ferroptosis in AD

Lipid metabolism dysregulation is a major pathway driving inflammation, synaptic loss, and memory deficits (Kao et al., 2020). Fatty acids influence membrane stability, signaling, and ion channels, modulating neuroinflammatory progression and AD pathogenesis (Cutler et al., 2004; Godos et al., 2020). For example, arachidonic acid (AA) promotes interleukin-6 (IL-6), IL-1, and leukotriene (LT) production (Schmitz and Ecker, 2008), all risk factors for AD. Due to its high lipid content and energy demands, the brain is prone to peroxidation of polyunsaturated fatty acids (PUFAs) (Petrovic et al., 2020; Sun et al., 2020). Clinical studies (Ferre-Gonzalez et al., 2022; Pena-Bautista et al., 2020, 2019) show elevated fatty acid peroxidation in AD brains. 4-hydroxy-2-nonenal (4-HNE), a toxic byproduct of fatty acid peroxidation, stimulates macrophages to trigger inflammation and AD progression, detectable across all AD stages (Renuka Sanotra et al., 2022; Yamashima, 2023). Astrocytes primarily mediate cerebral fatty acid catabolism, closely linked to APOE. APOE4 expression increases unsaturated triglyceride-rich lipid droplet formation in astrocytic endoplasmic reticulum (ER), impairing lipid droplet clearance and heightening sensitivity to lipid peroxidation, thereby elevating AD risk (Windham et al., 2024). A study (Bell et al., 2012) indicates that APOE4 independently triggers inflammatory cascades, causing neurovascular dysfunction, while APOE3 ameliorates this condition (Ioannou et al., 2019). Inhibiting APOE4 or enhancing APOE3 expression to promote FAO is an effective strategy to modulate ferroptosis and improve AD inflammation.

ROS are central to neuroinflammation. ROS accumulation triggers OS, cellular damage, mitochondrial dysfunction, Aβ deposition, Tau hyperphosphorylation, synaptic loss, and neuronal death—key factors in AD progression (Aliev et al., 2004; Islam, 2017; Nasb et al., 2024). Chen M. et al. (2023) found that anti-OS cerebroprotein hydrolysates limit Aβ accumulation, reduce p-Tau/Tau ratios, alleviate neuronal damage, and improve learning/memory in APP/PS1 mice. In the presence of free iron, Aβ actively participates in ROS generation, causing DNA damage, protein oxidation, and cerebral lipid peroxidation (Smith et al., 2007). ROS also activate microglia and astrocytes via p38MAPK/NF-κB and JAK2/STAT3 pathways, inducing pro-inflammatory cytokine production and disrupting the neuronal microenvironment, exacerbating OS and cell death (Linnerbauer et al., 2020; Xu et al., 2019). Microglial metabolic shifts toward anaerobic glycolysis and FAO influence immunophysiology, further increasing ROS and neuroinflammatory burden (Bogie et al., 2020; Pan et al., 2019).

Autophagy-dependent ferroptosis may underlie neuroinflammation in AD. A study (Houtman et al., 2019) shows that BECN1 deficiency enhances IL-1β and IL-18 release from microglia, causing aberrant neuroinflammation and neurodegeneration, potentially linked to high mobility group box 1 (HMGB1) secretion. HMGB1, actively secreted by inflammatory cells or passively released by necrotic cells, is critical for microglia-mediated neurotoxicity induced by Aβ and α-synuclein (Salminen et al., 2013; Song J. X. et al., 2014). HMGB1 upregulates TFR1 expression via Ras-JNK/p38 pathways, promoting ferroptosis (Ye et al., 2019). Normally, BECN1 interacts with the vacuolar protein sorting complex (Vps34) (Angelopoulou et al., 2018). Rotenone, a mitochondrial complex I inhibitor, induces HMGB1 overexpression, disrupting BECN1-Vps34 complex formation and impairing autophagy (Huang et al., 2017). Autophagy inhibition increases APP, Aβ, and α-synuclein aggregates, worsening memory deficits (Spencer et al., 2009; Yang et al., 2011). HMGB1 also activates NOX and NF-κB in microglia, inducing neurotoxic and pro-inflammatory molecule production (Gao et al., 2011). Targeting HMGB1 suppresses neuroinflammation, reduces Aβ production, and improves AD symptoms (Paudel et al., 2020). CMA may also contribute to AD neuroinflammation (Huang and Wang, 2025). Inhibiting p38MAPK reduces α-synuclein-induced neuroinflammation, particularly NLRP3 inflammasome activation, while increasing CMA receptor lysosomal-associated membrane protein 2A (LAMP2A) levels to enhance NLRP3 degradation (Chen et al., 2021a). Transcription factor EB (TFEB), a master regulator of the autophagy-lysosomal pathway, downregulates NLRP2 inflammasome via CMA activation (Song et al., 2021). However, a study (Wu et al., 2019) suggests that erastin-induced ferroptosis increases LAMP2A levels to activate CMA, which promotes GPX4 degradation and exacerbates ferroptosis. Key regulators like BECN1, HMGB1, and p38MAPK—shared by autophagy and ferroptosis—play pivotal roles in AD pathogenesis. Whether these molecules can serve as therapeutic targets for modulating ferroptosis and autophagy to alleviate neuroinflammation requires further.

#### 4.3.3 Relationship between gut microbiota dysbiosis and ferroptosis in AD

**Figure 8:**
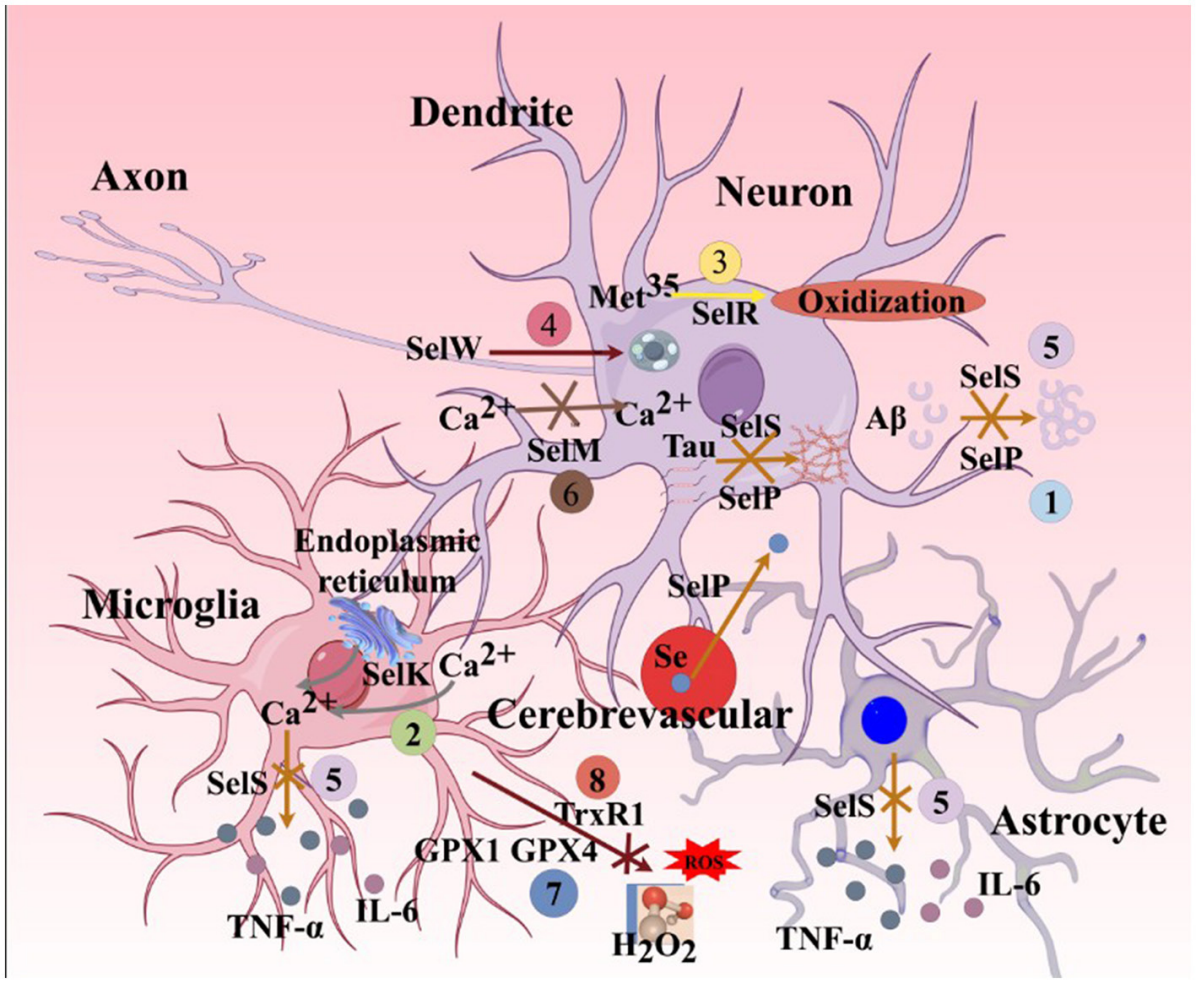
Major functions of selenoproteins associated with AD ferroptosis mechanisms. (1). SelP can promote the transport of selenium from cerebral blood vessels to brain parenchymal tissues. Its deficiency leads to a lack of selenium and various selenoproteins in brain parenchymal tissues. SelP also has the ability to inhibit the aggregation of Aβ and Tau proteins, thereby inhibiting ferroptosis. (2). SelK can promote the release of Ca^2^^+^ from the endoplasmic reticulum and further induce the influx of extracellular Ca^2^^+^, enhancing the migration and phagocytic abilities of microglia. (3). SelR has the function of preventing the oxidation of the amino acid residue Met35 in MSRA by ROS. (4). SelW primarily acts on the mTORC2/Akt signaling pathway, promoting autophagy in neurons. (5). SelS can reduce the release of L-6 from astrocytes, degrade Aβ in neurons, inhibit Tau protein phosphorylation caused by endoplasmic reticulum stress, and decrease the release of IL-6 and TNF-α in microglia. (6). SelM has the function of regulating calcium homeostasis in neurons. (7). GPX4 and GPX1 can inhibit the production of H_2_O_2_ and organic peroxides in neurons, astrocytes, and microglia. (8). TRXR1 can inhibit the production of H_2_O_2_ and organic peroxides in neurons. SelP, selenoprotein P; SelK,selenoprotein K; SelR,selenoprotein R; SelW, selenoprotein W; SelS, selenoprotein S; SelM, selenoprotein M; GPX1, glutathione peroxidase 1; MSRA, methionine sulfoxide reductase A; mTORC2, mammalian target of rapamycin complex 2; Akt, protein kinase B.

Gut microbiota produce neurotransmitters and neuromodulators, influencing immunity, brain development, and behavior. Human gut microbiota may act as a “second brain,” linked to neurodegenerative diseases like AD (Sochocka et al., 2019). Dysbiosis of gut microbiota and the gut-brain axis increases intestinal barrier permeability, allowing pathogens and neuroactive products to invade the nervous system, triggering cerebral neuroinflammation. The ratio of Bacteroides to Alistipes exhibited significant correlations with iron-related parameters. Compared to the normal iron group, both the iron-deficient and iron-overloaded groups showed declining trends in nucleotide metabolism, enzyme metabolism, and metabolic disease-related indicators, while lipid metabolism levels were markedly elevated (Long and Holtzman, 2019). Se improves gut ecology and enhances neuroactive substance levels via the “microbiota-gut-brain axis,” potentially delaying or ameliorating AD (Fu et al., 2024; Zhang Z. H. et al., 2017). In APP/PS1 transgenic mice, gut microbiota alterations correlate with enriched inflammatory bacterial taxa, mitigating amyloidosis and plaque-localized neuroinflammation (Chen et al., 2020). As a critical component of GPX4, Se modulates GPX4 expression in AD. Studies (Chen M. et al., 2024; Guo et al., 2021) show that Se reduces neuroinflammation, delays neurodegeneration, protects cognition, and reverses motor deficits. Adequate Se upregulates GPX4, attenuating lipid peroxide-mediated neuroinflammation in AD (Zhang and Song, 2021). The mechanisms of Se and selenoproteins in AD ferroptosis are complex, with cross-interactions among selenoproteins (details in Figure 8). Current research focuses on cellular and animal models, while human studies on Se-enriched foods or compounds for AD ferroptosis prevention remain limited and warrant further exploration.

### 4.4 Relationship between oxidative stress and ferroptosis in AD

#### 4.4.1 Relationship between mitochondrial oxidative stress abnormalities and ferroptosis in AD

OS is a shared pathological feature of ferroptosis and AD. While ROS originate from multiple sources, mitochondria are the largest contributors (Holmstrom and Finkel, 2014; Ray et al., 2012). P53 directly regulates cellular metabolic shifts by promoting mitochondrial oxidative phosphorylation (OXPHOS), increasing endogenous ROS production and acting as a positive regulator of ferroptosis-associated OS. Postmortem studies (Nelson and Xu, 2023) reveal elevated OS and p53 phosphorylation in AD brains, suppressing DNA damage response (DDR) and double-strand break (DSB) repair—potentially contributing to neuronal loss and offering new therapeutic targets. Mitochondria are the sole sites of heme synthesis and primary locations for Fe-S cluster assembly (Dutt et al., 2022; Ward and Cloonan, 2019). Iron-mediated oxidative damage increases heme levels and the pro-oxidant effects of iron released during HO-1-mediated heme degradation (Abraham et al., 2016). Mitochondrial destabilization disrupts redox balance. AD patients exhibit reduced intact mitochondria, impaired electron transport chain (ETC) enzymes, and dysfunctional OXPHOS and ETC, leading to neuronal hypometabolism (Perez Ortiz and Swerdlow, 2019). Dysfunctional ETC promotes APP hydrolysis, triggering ROS accumulation and OS exacerbation (Gabuzda et al., 1994). ROS interact with Fe-S clusters, catalyzing mitochondrial Fenton reactions to generate more ROS. Thus, mitochondrial iron's high ROS-generating propensity makes mitochondria ideal sites for AD ferroptosis execution.

Mitochondria are the main production sites of endogenous ROS, so it is feasible to intervene mitochondrial OS in the targeted treatment of AD. In forward electron transport, the respiratory chain transfers electrons from complex I to III via coenzyme Q (CoQ) (Scialo et al., 2020). Reverse electron transport (RET) drives electrons from complex II to I via CoQ, reducing NAD^+^ to NADH and increasing NADH/NAD^+^ ratios (Kowalczyk et al., 2021). This highly oxidized state of mitochondria greatly increases the production of O2- and mitochondrial ROS levels. CoQ10, as an electron carrier in the ETC and an endogenous lipophilic antioxidant, can reduce the expression of CoQ10 and increase its regeneration rate, thereby alleviating cystine deprivation-induced mitochondrial membrane potential hyperpolarization, lipid peroxidation, and ferroptosiss. In addition, McLellan et al. (2003) used multi-photon imaging technology to image Aβ deposits in the brains of AD animals and found that fluorescence generated by free radicals distributed around starch plaques. This indicates that the formation of Aβ is closely related to free radicals. Because mitochondrial DNA is closer to the inner membrane of mitochondria, it is more vulnerable to damage by free radicals, and lacks histone protection and damage repair system, and is more prone to mutations than nDNA (Liao et al., 2022). A study (Krainz et al., 2016) found that a compound targeting mitochondrial nitrogen oxide free radicals can effectively inhibit iron death in a variety of cells, suggesting that mitochondrial lipid peroxidation plays a key role in iron death. Through the development of free radical related REDOX nanoparticles, ROS can be removed to reduce OS reaction and play a protective role against AD *in vivo* and *in vitro* (Obulesu and Jhansilakshmi, 2016).

#### 4.4.2 Relationship between GSH/GPX4 axis abnormalities and ferroptosis in AD

The GSH/GPX4 axis is a critical antioxidant component in ferroptosis. Recent studies (Luo et al., 2023; Ursini and Maiorino, 2020; Zhang et al., 2024) identify GPX4 as a key enzyme counteracting LPO in hippocampal neurons, closely linked to AD neurodegeneration. GPX4-knockout mice exhibit cognitive decline and neurodegeneration associated with increased lipid peroxidation and ERK1/2 activation—not calpain 1 (CAPN1)—indicating ferroptosis, not apoptosis, as the driver (Hambright et al., 2017). Thus, targeting upstream GPX4 pathways is essential for AD ferroptosis intervention. Clinical research (Charisis et al., 2021) associates high plasma GSH levels with reduced AD risk. In neurons, Cys (Seib et al., 2011) and NAD(P)H (Dong et al., 2019) kinetically regulate GSH synthesis. Modulating Cys and NAD(P)H metabolism influences GSH levels, thereby affecting ferroptosis and AD pathology. Shea and Remington (2015) found that direct antioxidant application (e.g., L-Cys, Se) enhances AD treatment efficacy. Studies (Conus et al., 2018; Rapado-Castro et al., 2017; Sepehrmanesh et al., 2018) show that N-acetylcysteine (NAC) increases GSH in psychiatric patients' brains and blood cells, improving cognition. As a precursor for L-Cys and GSH, NAC exerts potent antioxidant effects (Mokhtari et al., 2017). Clinical and animal trials (Andrade et al., 2020; Moreira et al., 2007; Shahidi et al., 2017) confirm NAC's ability to reduce OS, promote axogenesis, prevent Aβ-induced memory deficits, and inhibit Tau self-aggregation. Conversely, Aβ oligomers impair Cys uptake and GSH synthesis by suppressing excitatory amino acid transporter 3 (EAAT3) (Hodgson et al., 2013), worsening OS in AD brains. Thus, GSH/GPX4 axis dysfunction is a key pathogenic mechanism in AD.

NADPH, another upstream GSH regulator, is a fundamental reductant for LPO clearance and a ferroptosis sensitivity biomarker (Stockwell et al., 2017). In AD, NADPH oxidase (NOX) contributes to ROS production. Excessive NOX-driven NADPH consumption reduces GSH synthesis, exacerbating OS (Fragoso-Morales et al., 2021). Cys and NAD(P)H share complex interconversion. Dong et al. (2019) demonstrated that external Cys/CySS redox shifts restore oxidized free NADH in aged neurons to youthful levels, countering AD pathology and extending lifespan. Cerebral oxidative damage and antioxidant defense are highly susceptible to iron-induced imbalance (Cheignon et al., 2018). AD patients and models show low antioxidant enzyme levels, with the AD risk gene APOE4 exhibiting weak antioxidative capacity, failing to inhibit oxidative damage. APOE4 activates p38MAPK to promote APP processing, Aβ oligomer formation, and AD progression (Pires and Rego, 2023; Youssef et al., 2018).

#### 4.4.3 Relationship between FSP1/CoQ10 axis abnormalities and ferroptosis in AD

The FSP1/CoQ10 axis is a parallel ferroptosis suppression pathway independent of GSH/GPX4 (Doll et al., 2019). CoQ10, synthesized via Cys metabolism, directly reacts with peroxide radicals to scavenge free radicals and control lipid oxidation (Kaur et al., 2024). Even at low concentrations, CoQ10 strongly mitigates OS (Cooper et al., 2008). As an ATP rate-limiting enzyme, CoQ10 regulates redox reactions, protecting mtDNA and mitochondrial membranes from radical attack and modulating energy production rates (Wang et al., 2024). Animal studies show CoQ10′s neuroprotection in AD models via OS reduction (Dumont et al., 2011), Aβ toxicity mitigation (Yang et al., 2008), mitochondrial enzyme activity restoration (Singh and Kumar, 2015), and anti-inflammatory effects (Choi et al., 2012). As an oxidoreductase, FSP1 uses NAD(P)H to reduce CoQ10 to ubiquinol (CoQ10H2) on cell membranes, generating lipophilic radical-trapping antioxidants that suppress ROS and LPO, countering ferroptosis (Kraft et al., 2020; Venkatesh et al., 2020). Beyond mitochondrial and enzymatic ROS sources, bioactive metal dyshomeostasis may contribute to Aβ/Tau-mediated radical generation and OS (Beal, 2005; Greenough et al., 2013; Wang and Wang, 2017). Fe^2^^+^/3^+^-Aβ complexes produce ROS, confirmed *in vitro* (Kim and Lee, 2021). However, the mechanisms disrupting redox balance and OS origins in AD remain unclear, necessitating further exploration of their interplay with ferroptosis.

### 4.5 Relationship between mitochondrial dysfunction and ferroptosis in AD

#### 4.5.1 Relationship between mitochondrial energy metabolism abnormalities and ferroptosis in AD

Mounting evidence (Deng et al., 2020; Mirzaei et al., 2019; Yadav et al., 2022) implicates mitochondrial dysfunction in AD pathogenesis, making mitochondrial mechanisms a hotspot for therapeutic development. Mechanisms include mitochondrial energy metabolism, OS, Ca^2^^+^ homeostasis, and mitophagy (mitochondrial OS discussed earlier). Mitochondrial energy metabolism is crucial for AD progression. The human brain constitutes 2%−3% of body weight but consumes ~20% of oxygen, relying solely on ATP (Goldberg et al., 2020). Cells shifting metabolism from glycolysis to OXPHOS are prone to ferroptosis. Early AD shows suppressed glucose metabolism in the hippocampus and cortex (Gordon et al., 2018). Aging-induced mitochondrial dysfunction triggers compensatory OXPHOS upregulation to maintain cerebral function (Schwartz et al., 2020). Voltage-dependent anion channel (VDAC) opening mediates metabolite influx into mitochondria, increasing OXPHOS and ROS production, causing mitochondrial dysfunction (Wang et al., 2020). As AD progresses, mitochondrial-centric metabolism declines. Most ATP derives from OXPHOS via ETC and ATP synthase (Ji et al., 2022). AD patients exhibit fewer, dysfunctional mitochondria and downregulated OXPHOS genes, leaving neurons hypometabolic. Chronic cerebral energy deficiency induces neuronal death, ROS generation, and impaired neurotransmission (Watts et al., 2018). A study (Liang et al., 2008) reports reduced nuclear-encoded ETC subunit genes in late-stage AD posterior cingulate, hippocampal CA1, middle temporal gyrus, and entorhinal cortex. Impaired mitochondrial complexes I, III, and IV exacerbate energy deficits (Perez Ortiz and Swerdlow, 2019). Targeting ATP synthase or other complexes to restore ATP production, normalize energy metabolism, and inhibit OS may suppress ferroptosis.

#### 4.5.2 Relationship between mitochondrial calcium homeostasis abnormalities and ferroptosis in AD

**Figure 9:**
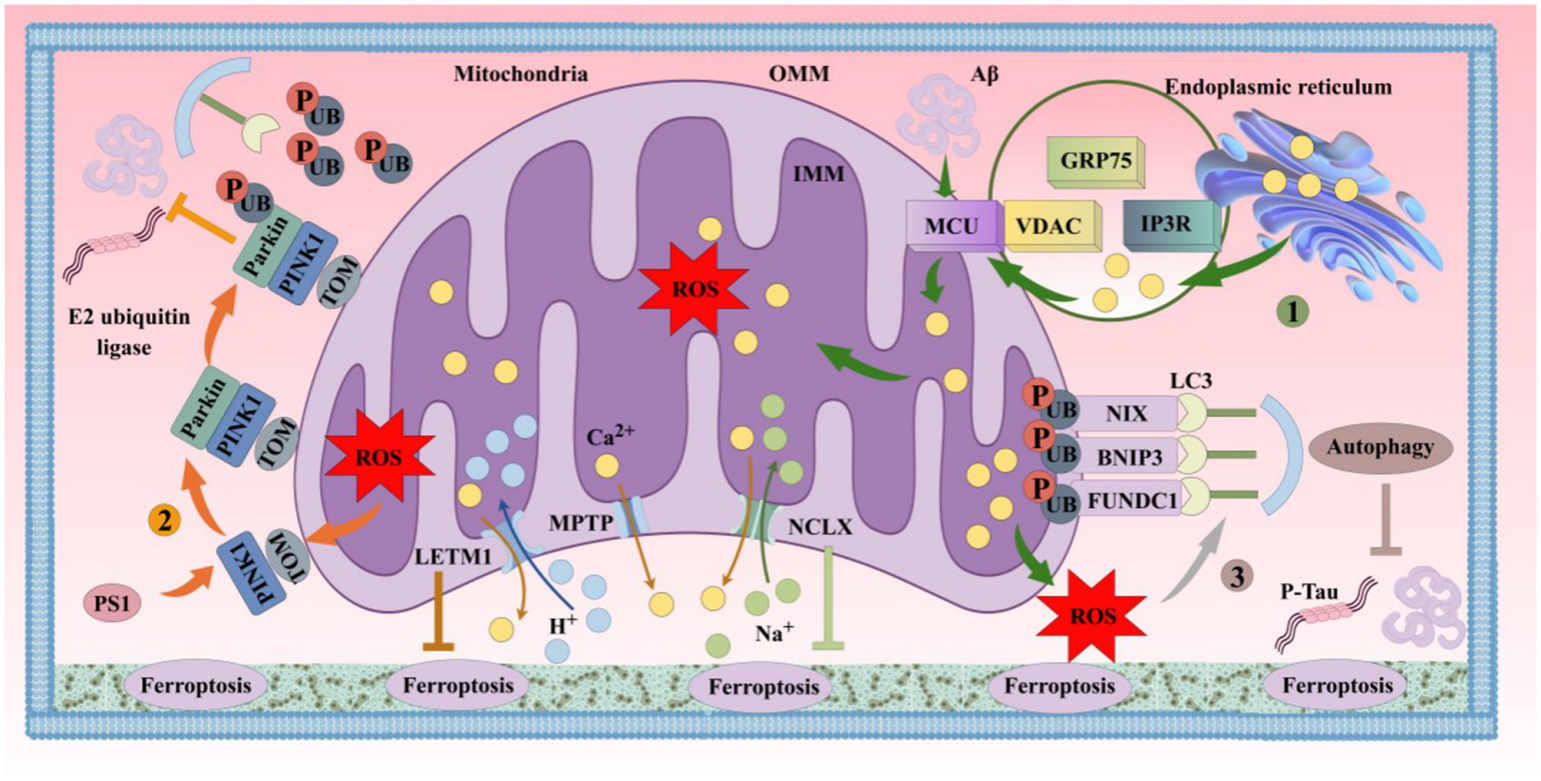
Schematic diagram of the mechanisms of mitochondrial Ca^2^^+^ homeostasis and mitophagy in regulating AD ferroptosis. (1). ER stress and Aβ promote mitochondrial Ca^2^^+^ influx, leading to mitochondrial Ca^2^^+^ overload, ferroptosis, and cellular dysfunction. (2). When mitochondrial damage reduces membrane potential, PINK1 accumulates on the outer mitochondrial membrane (OMM), forming a complex with TOM. PINK1 activates Parkin, which ubiquitinates mitochondrial proteins. Ubiquitinated mitochondria are engulfed by autophagosomes via LC3 complexes, fuse with lysosomes, and are degraded, inhibiting ferroptosis. (3). OMM receptors (e.g., BNIP3, NIX, FUNDC1) can directly bind LC3 to initiate mitophagy independently of the PINK1/Parkin pathway, suppressing ferroptosis. GRP75, glucose-regulated protein 75; IP3R, inositol 1,4,5-trisphosphate receptor; VDAC, voltage-dependent anion channel; MCU, mitochondrial calcium uniporter; OMM, outer mitochondrial membrane; IMM, inner mitochondrial membrane; PINK1, PTEN-induced putative kinase 1; Parkin/PARK2, Parkinson disease protein 2; TOM, translocase of outer mitochondrial membrane; LC3, microtubule-associated protein 1 light chain 3; BNIP3, BCL2/adenovirus E1B 19 kDa protein-interacting protein 3; NIX, BNIP3-like; FUNDC1, FUN14 domain-containing protein 1; LETM1, mitochondrial proton/calcium exchanger protein; MPTP, mitochondrial permeability transition pore; NCLX, mitochondrial sodium/calcium exchanger protein; PS1, presenilin 1.

Calcium ion homeostasis is fundamental to most biochemical reactions in cells. Calcium ions (Ca^2^^+^) are primarily stored in the endoplasmic reticulum (ER) and function through mitochondrial regulation to modulate basic cellular processes (Ryan et al., 2020). On one hand, mitochondrial Ca^2^^+^ uptake is mainly regulated by voltage-dependent anion channels (VDAC) on the outer mitochondrial membrane and the mitochondrial calcium uniporter (MCU) complex on the inner mitochondrial membrane. On the other hand, mitochondrial Ca^2^^+^ efflux occurs via the Na^+^/Ca^2^^+^/Li^+^ exchanger (NCLX) and Ca^2^^+^/H^+^ exchanger (LETM1) on the inner membrane (Calvo-Rodriguez and Bacskai, 2021; Datta and Jaiswal, 2021). Increased intracellular Ca^2^^+^ concentrations and excessive ROS production lead to elevated mitochondrial Ca^2^^+^ levels, mitochondrial fragmentation, and potential rupture of the mitochondrial membrane. Ruthenium red, a mitochondrial Ca^2^^+^ uptake inhibitor, can block ROS generation, indicating that mitochondrial Ca^2^^+^ contributes to ROS production. A study (Maher et al., 2018) has shown that reducing mitochondrial Ca^2^^+^ influx with compounds that counteract oxidative glutamate toxicity protects cells from ferroptosis induced by Xc^+^ system inhibitors. Jadiya et al. (2019) observed decreased expression of NCLX in the brains of AD patients and mice, leading to impaired mitochondrial Ca^2^^+^ efflux, mitochondrial Ca^2^^+^ overload, forced opening of the mitochondrial permeability transition pore (MPTP), neuronal death, and accelerated AD progression. Aβ aggregates enhance mitochondrial Ca^2^^+^ uptake by modulating MCU in MPTP, resulting in mitochondrial Ca^2^^+^ overload. This phenomenon can be prevented by the specific MCU inhibitor Ru360 (Calvo-Rodriguez et al., 2020). Restoring or enhancing NCLX or Ru360 expression promotes normal mitochondrial Ca^2^^+^ efflux, prevents mitochondrial Ca^2^^+^ overload and ferroptosis, and ameliorates AD pathology and cognitive deficits (Figure 9).

#### 4.5.3 Relationship between mitophagy abnormalities and ferroptosis in AD

Mitophagy, a selective autophagy process, is a critical cellular cleanup mechanism that maintains metabolic and cellular homeostasis by removing damaged mitochondria. Increased ROS production, altered mitochondrial permeability, and oxidative damage can induce mitophagy. The interplay between OS and mitophagy is also reflected in their association with stress-responsive proteins such as PI3K, Akt, mTOR, AMPK, JNK, and ERK (Dewanjee et al., 2022; Kim H. L. et al., 2023; Lan et al., 2017; Yang J. et al., 2022). Thus, mitophagy in AD is closely linked to ferroptosis. OS-induced mitochondrial damage in pyramidal neurons is considered a hallmark of mitophagy dysfunction in neurodegenerative diseases (Talebi et al., 2022). A study (Tedeschi and Secondo, 2022) identified transient receptor potential channel mucolipin-1 (TRPML1) as a sensor of intracellular ROS in neurons. Upon detecting elevated ROS levels, TRPML1 enhances neuronal mitophagy activity, accelerating the clearance of damaged mitochondria and reducing free radical production. Moderately increased ROS can act as signaling molecules to trigger mitophagy, restoring cellular homeostasis and inhibiting ferroptosis. However, abnormal mitophagy leads to the accumulation of dysfunctional mitochondria, exacerbating OS and Aβ generation, thereby worsening AD pathology (Roca-Agujetas et al., 2021; Sukhorukov et al., 2021) (Figure 9).

### 4.6 Relationship between cholinergic system abnormalities and ferroptosis in AD

#### 4.6.1 Relationship between abnormal acetylcholine synthesis/degradation and ferroptosis in AD

Cholinergic neurons are widely distributed in the human brain and play crucial roles in neurogenesis, neuronal differentiation, and synaptic plasticity (Long and Holtzman, 2019). Exogenous acetylcholine (ACh) administration or endogenous ACh release significantly improves long-term potentiation (LTP) and spatial memory in the mouse hippocampus (Kutlu and Gould, 2016). Conversely, hippocampal injection of scopolamine impairs memory formation and consolidation (Lee et al., 2014). Key enzymes in brain cholinergic systems include choline acetyltransferase (ChAT) and acetylcholinesterase (AChE). ChAT catalyzes the synthesis of ACh from choline (Ch) and acetyl-CoA (AcCoA), while AChE degrades ACh to terminate synaptic signaling. ChAT and AChE are linked to ferroptosis in AD pathology, likely through OS mechanisms. Intracerebroventricular streptozotocin (ICV-STZ) injection in rats induces hippocampal OS and neuronal damage (AD-TNDCI model) (Khan et al., 2012), reducing hippocampal ChAT activity and causing cholinergic deficits (Ishrat et al., 2006). Curcumin treatment in AD-TNDCI rats improves spatial learning/memory deficits, ChAT-positive neuronal loss, hippocampal OS, and neuronal damage (Ishrat et al., 2009). *In vitro* studies (Xi et al., 2011) show that the antioxidant genistein (Gen) alleviates Aβ25-35-induced mitochondrial membrane fluidity (MMF) decline, maintains mitochondrial membrane potential (MMP), enhances GSH/GSSG ratios and GPX activity, inhibits AChE mRNA expression, and boosts mitochondrial antioxidant capacity. Additionally, rotenone upregulates p53 and COX2, inhibits ChAT, GPX4, and SLC7A11 activity, induces ferroptosis and cholinergic dysfunction, and causes neuronal dysfunction (Huang et al., 2022). Nguyen et al. (2023) found that mountain-cultivated ginseng activates the Nrf2/ChAT/ERK pathway, protecting cognitive function in GPX1-knockout mice by modulating cholinergic parameters and redox processes. These findings suggest that ChAT- and AChE-positive neurons are closely associated with ferroptosis, but specific signaling pathways linking cholinergic system protection to ferroptosis inhibition require further exploration.

#### 4.6.2 Relationship between AChR abnormalities and ferroptosis in AD

In mouse models, AChR deficiency in the prefrontal cortex causes attention deficits, which are rescued by receptor expression restoration (Guillem et al., 2011). ACh binds to postsynaptic AChRs, including nicotinic (nAChR) and muscarinic (mAChR) subtypes, altering neuronal membrane potentials or activating intracellular signaling (Lombardo and Maskos, 2015). Studies (Ballinger et al., 2016) suggest that α7-nAChR and M1-mAChR-mediated cholinergic pathways are key mechanisms for ACh-regulated hippocampal LTP. Recent evidence highlights the critical role of AChRs (α7-nAChR, M1-mAChR) in modulating ferroptosis to improve AD cognitive dysfunction.

α7-nAChR inhibits microglial activation, shifting the M1 phenotype to the anti-inflammatory M2 phenotype, and ameliorates ferroptosis (Luo and Huang, 2024). Specific agonists of the α7-nAChR receptor can improve AD ferroptosis and cognitive impairment to a certain extent by inhibiting neuronal γ-secretase activity and promoting microglial phagocytosis of Aβ. In addition, dysfunction of the Xc- system in ferroptosis leads to increased Glu concentration, causing neuroexcitotoxicity. The excitotoxicity caused by the accumulation of a large amount of Glu and cholinergic dysfunction is one of the pathogenic mechanisms of AD (Muralidar et al., 2020). α7-nAChR can enhance synaptic transmission between hippocampal neurons by stabilizing the expression of GluA1 receptors on dendritic spines (Halff et al., 2014). However, α7-nAChR can also promote ferroptosis and exert cytotoxic effects. A study (Wu M. et al., 2021) showed that high concentrations of Aβ lead to a reduction in recombinant human lymphocyte antigen 6H (Ly6h), an increase in Ca2+ influx mediated by α7-nAChR, and increased Tau hyperphosphorylation and neurotoxicity in the brains of AD patients. At the same time, a bioactive 14-peptide (T14) from the C-terminus of AChE significantly increases in the early brains of AD patients (Ranglani et al., 2024). T14 plays a trophic role in early development through the calcium signaling of α7-nAChR, but the tolerance of neurons to calcium ion flux significantly decreases in the elderly (Graur et al., 2023). This process may shift to excitotoxicity when activated in the adult brain, becoming the basis of AD degeneration (Greenfield et al., 2022). The dual effect of α7-nAChR in promoting neurotrophic and neurotoxic effects may be due to the fact that α7-nAChR agonists exhibit “activation” at low concentrations but produce toxic effects and desensitization at high concentrations (Yang et al., 2017). Therefore, the activity of α7-nAChR should be maintained within an appropriate range to avoid ferroptosis and allow neurons and the brain to function normally.

In addition, M1-mAChR may play a key role in the process of inhibiting ferroptosis. The activation of Gq/11-coupled mAChRs (including M1) can protect cells from death induced by DNA damage, oxidative stress (OS), and mitochondrial dysfunction (Liu B. et al., 2022; Liu et al., 2020; Wu F. et al., 2021). M1-mAChR can also antagonize the neurotoxicity induced by Glu through the PKC-ERK 1/2 pathway, protecting neurons in the AD brain and improving cognitive levels and learning abilities (Ma et al., 2013). The key ferroptosis molecule GPX1 gene can also protect against memory impairments induced by Aβ (1–42) by activating the cAMP response element-binding protein/brain-derived neurotrophic factor (CREB/BDNF) signaling pathway dependent on M1-mAChR (Shin et al., 2021). Studies (Fisher et al., 2003; Gu et al., 2003) have shown that the activation of M1-mAChR promotes the α-secretase-mediated cleavage of APP, thereby inhibiting its cleavage by BACE-1 and γ-secretase to produce neurotoxic Aβ, and can significantly reduce Aβ excitatory neurotoxicity (action potential firing and excitatory postsynaptic currents). The above studies all indicate that AChR (α7-nAChR, M1-mAChR) function plays an important role in regulating ferroptosis and improving AD cognitive dysfunction.

#### 4.6.3 The relationship between other cholinergic neuron abnormalities in AD and ferroptosis

Cholinergic synaptic transmission is an important way for other neurons in the brain to communicate, such as glutamatergic (Glu) neurons and γ-aminobutyric acid (GABA)ergic neurons. Glutamate (Glu) is an important excitatory amino acid neurotransmitter, with the highest content in brain tissue, released by about 3% of synapses, and acts on postsynaptic membrane receptors, mainly the N-methyl-D-aspartate receptor subtype (NMDAR), to exert excitatory regulatory effects. When Glu release increases or reuptake is inhibited, continuous stimulation of NMDAR by Glu leads to massive calcium influx, resulting in excitotoxicity (Beas-Zarate et al., 2001). This is also one of the mechanisms of ferroptosis. Aβ can also directly increase ROS production by activating NOX and indirectly increase NMDAR by stimulating the release of arachidonic acid (AA) (Shelat et al., 2008). Moreover, increasing evidence shows that the GABA nervous system can regulate the activity of Glu and NMDAR-mediated glutamatergic nervous system, maintain the balance of excitation and inhibition in the nervous system, and thereby regulate the synaptic plasticity of neurons (Chiu et al., 2018; Gerhard et al., 2020). GABA is formed by the decarboxylation of Glu in the brain under the action of glutamate decarboxylase and is the most widely distributed inhibitory neurotransmitter in the central nervous system. The activation of GABA receptors on the neuronal cell membrane opens chloride ion channels, leading to chloride ion influx and the generation of inhibitory postsynaptic potentials, thereby inhibiting the excitation of postsynaptic neurons. A study (Liu C. et al., 2022) showed that by activating GABAB receptors and regulating the AKT/GSK 3 β/β-catenin signaling pathway, the expression of SLC7A11 and GPX4 could be increased, alleviating brain edema, OS, and BBB damage, and inhibiting neuronal ferroptosis. Although the specific relationship between the cholinergic system and AD ferroptosis is not clear, as research deepens, cholinesterase inhibitors, M1/M4 muscarinic receptor agonists, and presynaptic choline transporters are being developed, which may help alleviate the progression of AD ferroptosis and improve clinical symptoms such as cognitive disorders, memory impairments, and spatial disorientation.

### 4.7 The relationship between other pathogenic mechanisms of AD and ferroptosis

#### 4.7.1 The relationship between DNA methylation abnormalities in AD and ferroptosis

Genome-wide association studies (GWAS) have found that genetic factors play an important role in AD risk, with more than 40 genetic loci related to AD identified. In the late stages of AD, neurons and glial cells in the lesion areas show a large amount of chromatin relaxation, heterochromatin opening, and active region closure, a phenomenon known as the global loss of epigenetic information (Xiong et al., 2023). DNA methylation can regulate gene expression and is closely related to the occurrence of AD. A study (Di Francesco et al., 2015) showed that the expression level of DNA methyltransferase 1 (DNMT1) increased in AD patients over 65 years old and was related to the expression of the AD risk gene APOE4. Sun W. et al. (2020) knocked down the expression of DNMT1 and DNMT3a in primary cultured hippocampal neurons and found that hippocampal neurons exhibited fewer and thinner dendritic branches, lower density of excitatory synapse formation, and could inhibit Glu-induced Ca2+ influx. However, another study (Feng et al., 2010) showed that the lack of DNMT1 and DNMT3a led to defects in synaptic plasticity, learning, and memory in the hippocampal CA1 region. This may be related to different stages of AD and target sites. DNMT1 is also related to the occurrence of ferroptosis. DNMT1 can inhibit ferroptosis by activating the RUNX/p53 pathway and promoting the expression of SLC7A11. However, another study found that DNMT1 promoted ferroptosis by downregulating GPX4 expression and activating ferritin autophagy (Dong et al., 2022). The mechanisms by which DNMT1 targets ferroptosis in different diseases to induce or inhibit its occurrence need to be explored. However, there are currently no studies investigating the role of DNMT1 in regulating ferroptosis in AD. In addition, Huls et al. (2022) conducted an association study on brain DNA methylation and cognitive trajectory changes in 636 patients and found abnormal DNA methylation modifications in claudin 5 (CLDN5), an important protein for the BBB. An animal study (Liu et al., 2023) showed that downregulation of CLDN5 under hypoxic conditions could significantly activate the expression of ferroptosis-related genes and inhibit the expression of SLC7A11, inducing BBB disruption. This suggests that abnormal methylation of CLDN5 activating ferroptosis-induced BBB dysfunction may play an important role in AD. The above studies consistently point out that DNA methylation may play a crucial role in the regulation of ferroptosis mechanisms in AD. However, in-depth exploration in this area is still relatively scarce. Therefore, future research should increase the investigation of the relationship between DNA methylation and ferroptosis in AD to gain more detailed and in-depth insights.

#### 4.7.2 The relationship between histone modification abnormalities in AD and ferroptosis

Histone modifications, as an important component of epigenetics, are closely related to the occurrence and development of AD. The levels of histone modifications are regulated by multiple factors, and most modifications (such as methylation, acetylation, or ubiquitination) mainly occur on lysine or arginine residues (Zhang H. et al., 2020). Histone lysine methylation and acetylation usually act in a mutually antagonistic manner. One of the most studied lysine modifications is the methylation of histone H3 lysine 9 (H3K9). H3K9 mono- or dimethylation is involved in transcriptional repression, while trimethylation is involved in gene silencing (Black and Whetstine, 2011). H3K9 acetylation promotes the expression of active genes (Turner, 2000). A study (Habibi et al., 2011) found that methylation at the H3K9 site had a negative impact on gene expression. In a late-onset familial AD (FAD) mouse model, H3K9 dimethylation (H3K9me2) and histone methyltransferase 1 (EHMT1) and EHMT2 were significantly increased in the prefrontal cortex, a key cognitive region in AD (Zheng et al., 2019). Treatment with the selective EHMT inhibitor UNC0642 could reduce EHMT2 and H3K9me2 levels in AD models and reverse spatial and recognition memory deficits (Wang et al., 2021). Histone H3K9 demethylase KDM3B, as a potential epigenetic regulator of ferroptosis, can inhibit the occurrence of ferroptosis by upregulating the expression of SLC7A11 (Shinkai and Tachibana, 2011; Wang Y. et al., 2020). Therefore, H3K9me may be a key molecule regulating the occurrence of AD ferroptosis.

Changes in the dynamic balance between histone acetyltransferase (HAT) and histone deacetylase (HDAC) can lead to reduced H3 acetylation in the mouse brain, inhibiting the transcriptional activation of genes related to neuroplasticity and resulting in cognitive impairments in mice (Aggarwal et al., 2020). Nativio et al. (2020) found through RNA sequencing that H3K9ac modification was increased in the brains of AD patients, and HAT-related gene expression was upregulated. They also found that increased H3K9ac in AD fruit fly models promoted Aβ1-42-induced neurodegeneration, confirming the negative effects of increased acetylation. This indicates that there are differences in the results of histone modification markers in AD studies. Such differences may be due to different AD subtypes, disease stages, and experimental methods, and also reflect the complexity of the histone acetylation process in AD. HDAC (SIRT1) activation can exert therapeutic effects on age-related diseases such as AD by targeting H3K9 acetylation (Cui et al., 2022). A study (Wen et al., 2024) also showed that reducing the downregulation of the SIRT1/Nrf2/GPX4 pathway could increase the expression of SLC7A11 and inhibit ferroptosis in the hippocampus of splenectomized rats, thereby reducing the negative impact on cognitive function. In addition, histone deacetylase inhibitors can also inhibit the accumulation of lipid ROS by suppressing the expression of NOX genes (Chen et al., 2016; Pandey et al., 2012). This indicates that NOX acetylation can inhibit ferroptosis through the lipid ROS pathway. Sequencing studies on the acetylation of the olfactory cortex in the brains of AD patients have identified more than 4,000 differential acetylation sites, with related genes including APP, PS1, PS2, and MAPT (Marzi et al., 2018). However, it is worth noting that previous AD studies have not identified abnormal epigenetic activation as a functional pathway for regulating AD ferroptosis, possibly due to the lack of comparison between disease states and healthy brain aging. These studies suggest that histone modifications may be a potential epigenetic target for AD ferroptosis treatment, but further validation is needed.

#### 4.7.3 The relationship between abnormal non-coding RNA regulation in AD and ferroptosis

To date, more than 100 types of modifications have been found in mammalian miRNAs, tRNAs, rRNAs, and lncRNAs (Li and Mason, 2014). Among them, N6-methyladenosine (m6A) methylation is the most common modification in eukaryotes. A study (Castro-Hernandez et al., 2023) showed that the level of m6A methylation in the brains of AD patients was lower than that in cognitively intact elderly individuals. In AD mouse models, the m6A methylation level of transcripts related to disease progression decreased, and the corresponding protein expression levels also decreased (Shafik et al., 2021). In contrast, the m6A demethylase FTO promotes the degradation of Olig2 mRNA, reducing myelin production in the mouse brain. Mice with the FTO gene knocked out showed increased m6A levels in the brain and improved memory consolidation (Xia et al., 2023). Additionally, overexpression of FTO in neurons can increase Tau protein phosphorylation through mTOR signaling (Li et al., 2018). However, a high-throughput sequencing result showed that m6A modification levels in AD mouse genes generally increased, with increased expression of the m6A methyltransferase METTL3 and decreased expression of the m6A demethylase FTO (Han et al., 2020). Another study (Zhang and Gong, 2024) indicated that overexpression of FTO could downregulate the m6A level of FYN mRNA, thereby downregulating the expression of the FYN protein, a member of the Src kinase family, and inhibiting neuronal ferroptosis. The activation of FYN can lead to the accumulation of Aβ plaques in the brains of AD patients, abnormal phosphorylation of Tau protein, and neuronal damage, resulting in a gradual decline in cognitive function and memory (Meur and Karati, 2025). This suggests that both excessive inhibition and activation of m6A methylation can lead to neuro damage, which may be related to different stages of AD onset and different cell types and states.

The significant increase in m6A-modified genes may also lead to the conversion of microglia to the M1 phenotype and involvement in inflammatory responses (Li Q. et al., 2021). As mentioned earlier, iron and iron chelators play an important role in the balance between M1 and M2 phenotypes, and regulating m6A methylation may affect the process of ferroptosis. In addition, according to bioinformatics data, the ferroptosis-related factor APOE4 may be closely related to m6A methylation regulators in the brain tissue of AD patients (METTL3, METTL16, YTHDC2, and LRPPRC) (Du et al., 2021). The above research results suggest that m6A modification may be closely related to the occurrence and development of ferroptosis in AD and requires further in-depth research for verification. However, the majority of genetic risk loci are located in the non-coding regions of the genome, and each locus has a small individual contribution to the disease phenotype, making precise localization of disease sites and mechanism parsing for AD a huge challenge (Bellenguez et al., 2022; Ridge et al., 2013; Schwartzentruber et al., 2021). Solving these problems requires single-cell level association analysis of epigenomes and transcriptomes in AD to study the molecular biological maps of AD at different stages of onset in different cell types and states.

## 5 Conclusion

In recent years, with the acceleration of population aging, the number of AD patients has been increasing year by year. The development and use of drugs for AD still need to be further explored, which requires the identification of new targets for in-depth research. Ferroptosis, as a newly discovered form of cell death, has attracted the attention of many scholars. As research continues to deepen, more experimental evidence indicates that ferroptosis has a significant impact on the progression of AD. Although the specific mechanisms underlying ferroptosis are not yet clear, and the role of ferroptosis in the pathological process of AD still needs to be investigated, considering the biological regulatory mechanisms of ferroptosis, the pathogenesis of AD, the uniqueness and importance of iron in AD, and the close relationship between ferroptosis and AD, targeting ferroptosis will become a new therapeutic target for AD. By analyzing the distinct hallmarks of ferroptosis, this study comprehensively elucidates its involvement in AD pathogenesis and underlying mechanisms, successfully establishing connections between fundamental biological processes and innovative intervention strategies, with the goal of advancing targeted therapeutic development for AD.

## 6 Limitation and Future perspective

Although current research has revealed the critical role of ferroptosis in the pathological progression of AD, several limitations remain to be addressed in this field: (1) Existing evidence primarily relies on animal models or *in vitro* cellular experiments, failing to comprehensively reflect the spatiotemporal dynamics of iron metabolism dysregulation in the brains of human AD patients. (2) The causal relationship between ferroptosis and classical AD pathological hallmarks such as Aβ deposition and hyperphosphorylated tau protein remains unclear. Particularly, how iron overload influences the neuroinflammatory microenvironment through epigenetic modifications (e.g., DNA/RNA methylation) requires validation through the establishment of a multi-omics integrative analysis framework. (3) Current ferroptosis inhibitors (e.g., Ferrostatin-1) exhibit limitations such as low blood-brain barrier penetration and poor tissue specificity, while clinically used iron chelators (e.g., deferoxamine) may interfere with physiological iron homeostasis. There is an urgent need to develop neuron-targeted nanoparticle-based drug delivery systems. Ferroptosis is more likely to act as a crucial node rather than an independent pathway within the complex pathological network of AD.

Future research should strengthen interdisciplinary collaboration by incorporating ferroptosis theory into the systems biology research paradigm of AD, ultimately achieving closed-loop breakthroughs from mechanistic investigations to clinical translation. In summary, future studies will deepen our understanding of ferroptosis, thereby providing new perspectives for combating neurodegeneration and brain aging in AD.
